# Demonstration of Controlled Skyrmion Injection Across a Thickness Step

**DOI:** 10.1021/acs.nanolett.4c01605

**Published:** 2024-05-23

**Authors:** Matthew T. Littlehales, Samuel H. Moody, Luke A. Turnbull, Benjamin M. Huddart, Ben A. Brereton, Geetha Balakrishnan, Raymond Fan, Paul Steadman, Peter D. Hatton, Murray N. Wilson

**Affiliations:** † Durham University, Department of Physics, South Road, Durham, DH1 3LE, United Kingdom; ‡ ISIS Neutron and Muon Source, Rutherford Appleton Laboratory, Didcot, OX11 0QX, United Kingdom; § Laboratory for Neutron Scattering and Imaging, Paul Scherrer Institute, Villigen, CH-5232, Switzerland; ∥ Max Planck Institute for Chemical Physics of Solids, Noethnitzer Str. 40, 01187 Dresden, Germany; ⊥ Department of Physics, Clarendon Laboratory, University of Oxford, Parks Road, Oxford, OX1 3PU, United Kingdom; # University of Warwick, Department of Physics, Coventry, CV4 7AL, United Kingdom; ○ Diamond Light Source, Didcot, OX11 0DE, United Kingdom; □ Memorial University of Newfoundland, Department of Physics and Physical Oceanography, St John’s, Newfoundland, A1B 3X7, Canada

**Keywords:** magnetic skyrmions, small-angle X-ray scattering, 3D magnetic nanostructures

## Abstract

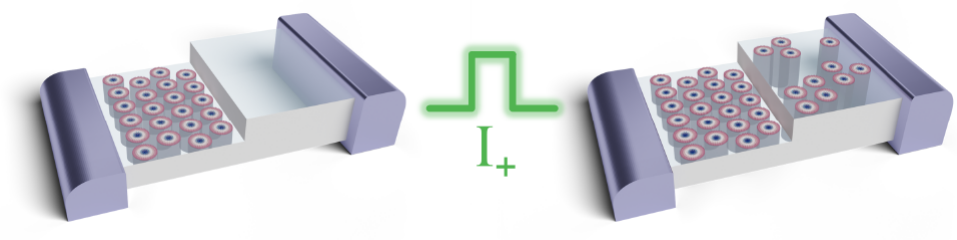

Spintronic devices incorporating magnetic skyrmions have attracted significant interest recently. Such devices traditionally focus on controlling magnetic textures in 2D thin films. However, enhanced performance of spintronic properties through the exploitation of higher dimensionalities motivates the investigation of variable-thickness skyrmion devices. We report the demonstration of a skyrmion injection mechanism that utilizes charge currents to drive skyrmions across a thickness step and, consequently, a metastability barrier. Our measurements show that under certain temperature and field conditions skyrmions can be reversibly injected from a thin region of an FeGe lamella, where they exist as an equilibrium state, into a thicker region, where they can only persist as a metastable state. This injection is achieved with a current density of 3 × 10^8^ A m^–2^, nearly 3 orders of magnitude lower than required to move magnetic domain walls. This highlights the possibility to use such an element as a skyrmion source/drain within future spintronic devices.

An enormous amount of attention continues to focus on magnetic skyrmions, vortex-like spin configurations whose spins completely wrap the unit sphere.^1−4^ Their particle-like character and emergent electrodynamics cause a number of exotic physical properties, including the topological Hall effect (THE), a result of the emergent magnetic field of the 3D noncoplanar spin configuration.^5^ Consequently, skyrmions can be controlled by a number of low-energy methods.^6−11^ However, most importantly, their coupling with conduction electrons leads to the efficient motion of the magnetic skyrmion via spin transfer torque (STT) with threshold current densities often 5 orders of magnitude smaller than conventional domain wall devices.^12,13^

In noncentrosymmetric materials,^3,14−16^ the origin of the magnetic skyrmion lies in the Dzyaloshinskii–Moriya interaction, a result of broken inversion symmetry.^17^ The competing symmetric and antisymmetric exchanges prefer parallel and perpendicular spin alignments, respectively, resulting in helical ground states and a hexagonal Bloch type skyrmion lattice. Bloch skyrmions have a well-known 2D spin configuration consisting of a transverse winding of the magnetization from an up/down core, antiparallel to the magnetic field, to a polarized background, and are characterized by an integer winding number defined by
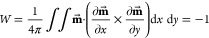
1where  is the unit magnetization. The skyrmion then permeates through the bulk to form an extended tube^18−20^ and terminates at either a surface or through the formation of a Bloch point singularity.^21^

Within bulk crystals, the skyrmion lattice in equilibrium is limited to a window of finite magnetic fields just below the magnetic ordering temperature, *T*_C_, owing to thermal fluctuations which favor the skyrmion over the competing conical phase.^3^ In contrast, metastable skyrmions with effectively infinite lifetimes can exist in local energy minima, and have previously been formed via rapid field cooling.^22−24^ However, as the system dimensionality reduces, thereby confining the skyrmion lattice along its third dimension, its stability increases relative to the conical phase, allowing the equilibrium skyrmion lattice to exist at much lower temperatures and significantly higher magnetic fields,^14^ providing a degree of tunability. In addition, their lifetimes in thin films are notably longer than in bulk single crystals.^25^

The effects of constrained geometries and low dimensionalities on skyrmions have already been extensively studied,^26−29^ and more exotic textures have subsequently been realized.^30−33^ Therefore, with skyrmions and higher order topological objects in mind, the recent developments in 3D nanostructure fabrication provides real prospects in developing enhanced spintronic devices,^34^ including a number of concepts already realized.^35,36^

It is with these concepts in mind that we experimentally demonstrate current induced skyrmion motion across a stepped FeGe lamella. Utilizing magnetically sensitive, resonant small-angle X-ray scattering (SAXS), we find separation in the equilibrium *B*–*T* conditions for skyrmion formation between the two sample thicknesses, and by measuring SAXS patterns from the thicker region, we demonstrate the ability to reversibly move skyrmions across the step with a current density of 3.83 × 10^8^ A m^–2^. We demonstrate a practical method of skyrmion nucleation and highlight the potential of future three-dimensional device regimes, motivating further exploration into the dynamics of skyrmions within complex geometries.

With skyrmion stability known to be highly dependent on sample thickness,^14^ a question arises when two sections of a device are different thicknesses, separated by a step. Can we move the skyrmions across this barrier into a regime of metastability? To test this hypothesis, we fabricated a “skyrmion injector” device from a single crystal of FeGe with two regions of thicknesses 300 and 500 nm separated by a step-like thickness barrier. FeGe, the prototypical metallic skyrmion host, provides a unique opportunity to study dynamical skyrmion mechanisms close to room temperature, without suffering from the prevalent disorder present in CoZnMn.^37^ The sample thicknesses were chosen to maintain the equilibrium skyrmion conditions close to room temperature, therefore replicating, within the limitations of the material, the desired working conditions of a skyrmionic device. Consequently, a penetrating X-ray technique, such as SAXS, is required to observe the injection mechanism, since Lorentz transmission electron microscopy (LTEM) works only with thicknesses less than ≈100 nm. We fixed the stepped lamella to a Au-backed Si_3_N_4_ membrane over a 4.5 μm aperture (with Pt deposition) such that the X-rays sampled only the thick region of the device. Electrical currents were subsequently applied along the long axis of the lamella via sputtered AuPd electrodes (see Supporting Information (SI) for further details^38^). In addition, we fabricated a second sample from the same 300 nm thick region of the lamella and fixed it to a separate aperture without current contacts. A schematic of both samples (insets) is shown in Figure 1a. Consequently, we measured the SAXS signal from two separate lamellae, sampling the two regions independently. Both phase diagrams were measured by zero field cooling (ZFC) from 290 K to the desired temperature, and measuring signals of helix, conical, or polarized, and skyrmion phases (Figure 1b–d) as a function of increasing field. Example SAXS patterns are found in Figure 1e–g. We present the dual thickness *B*–*T* phase diagram in Figure 2a, and indicate a region in which skyrmions exist only within the 300 nm section (star and circle fill) and no magnetic contrast is present in the 500 nm sample. This region is therefore termed as the suitable “injection condition”, where skyrmions are only present within the thinner region of the device.

**Figure 1:**
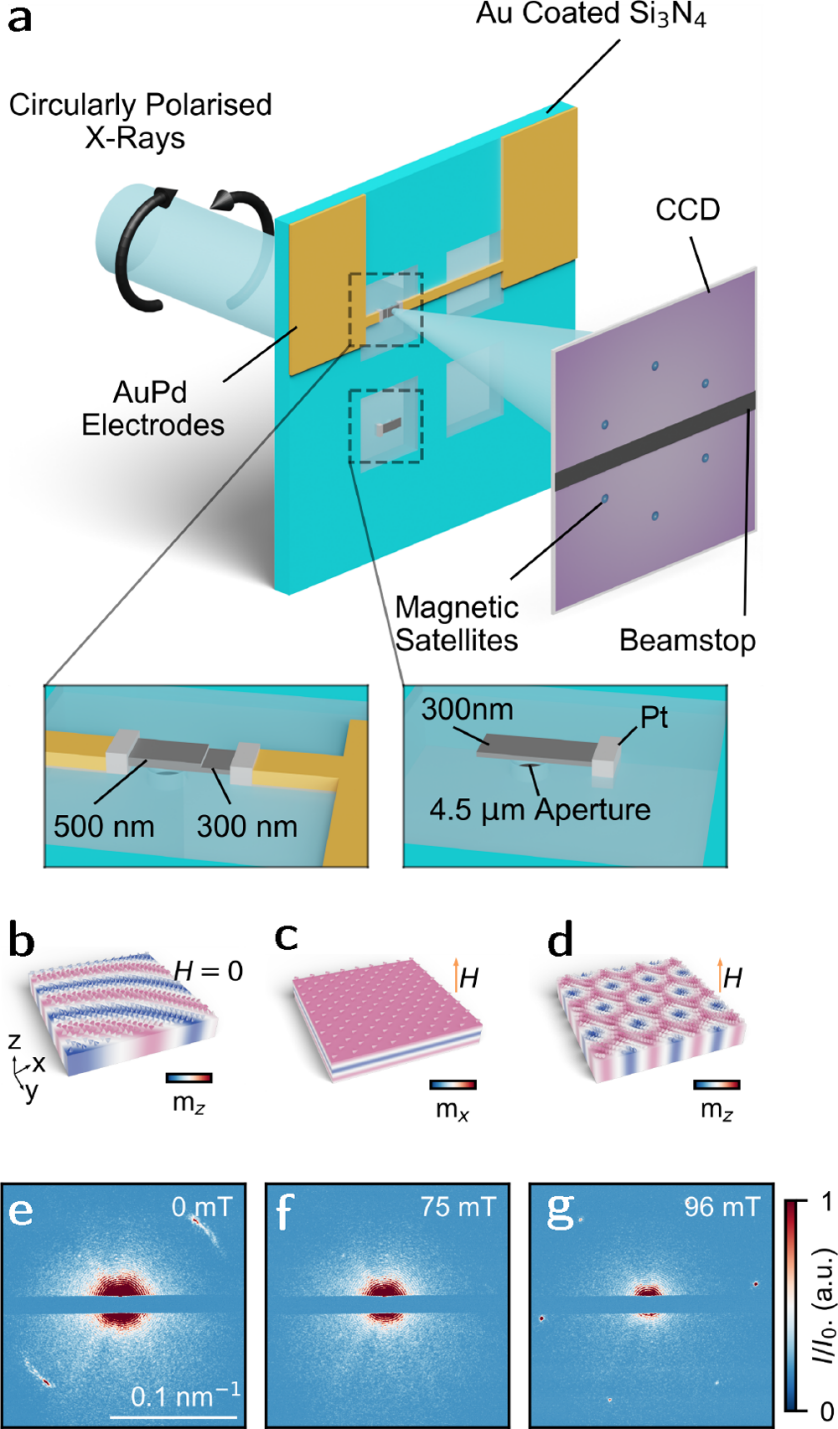
(a) Schematic of SAXS measurement process with insets corresponding to the two sample geometries measured within this study. (b–d) Schematics of helix, cone, and skyrmion states and corresponding SAXS scattering patterns (e–g) taken at the Fe L_3_ absorption edge from the 500 nm thick region.

**Figure 2:**
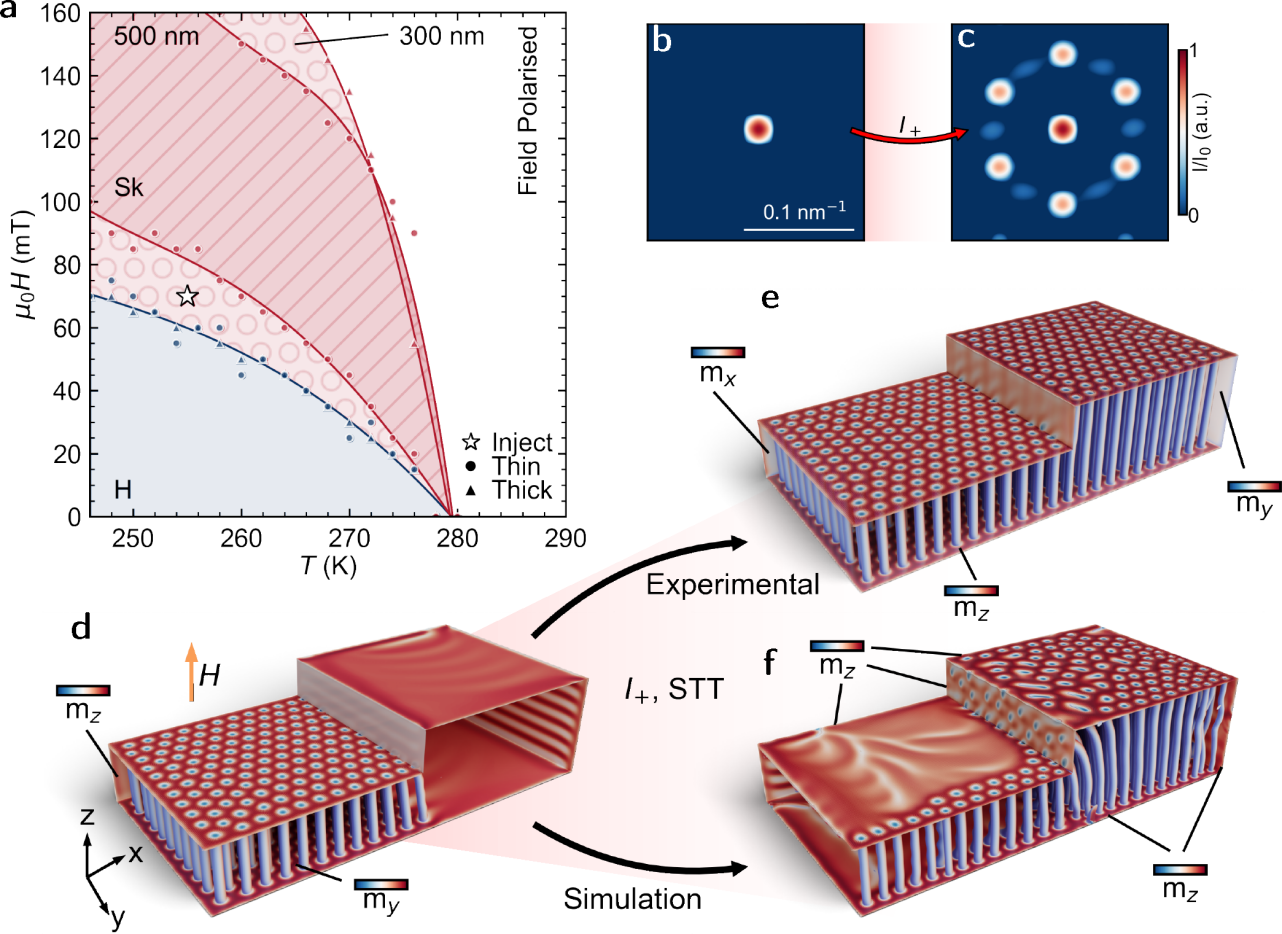
Magnetic phase diagram and micromagnetic simulations: (a) Magnetic phase diagram measured using SAXS after ZFC from 300 K to a chosen temperature between 245 and 280 K and measuring scattering patterns at 5 mT intervals from 0–160 mT. Blue region corresponds to the helix phase (H), and red corresponds to the skyrmion phase (Sk). The hatched region denotes the skyrmion pocket from the 500 nm region of the device, and the filled circle corresponds to the injection condition where there is a skyrmion lattice present in the 300 nm region and a conical phase/polarized phase in the 500 nm region. The star indicates the conditions where measurements in Figures 3, 4, and 5 were performed. (b, c) Simulated reciprocal space scattering patterns of the thick region before and after injection, corresponding to the thicker section of (d) and (e). (d, e) Micromagnetic simulations of skyrmion configuration when equilibrium conditions are satisfied for only the thin region (d) and for both regions (e). (e) also demonstrates the expected state after a current pulse, as inferred from the experimental results. (f) Simulated final state after a current pulse based on the starting state (d).

To visually demonstrate the expected behavior of our sample in this injection region, we present micromagnetic simulations. Using a system size of 2 μm × 1 μm with a 300 nm thin region and a 500 nm thick region, the simulations balance reasonable computation time with size approaching experimental conditions. For these example images in Figure 2d,e, we manually nucleated an approximation of the expected state and then relaxed the micromagnetic energy. Figure 2d shows the expected magnetization before any current pulse is applied; this consists of a highly ordered skyrmion lattice in just the thin region, with a conical state in the thick region (the skyrmion state in these *B*–*T* conditions is only the equilibrium state in the thin region). After applying a sufficient current pulse of positive polarity (from thick region toward the thin region), we expect skyrmions to be injected into the thick region (with new skyrmions renucleated in the thin region). This would result in a state, as shown in Figure 2e, where we have a reasonably well-ordered skyrmion lattice throughout the sample. Fourier transforms of the *z*-component of the magnetization in the thick region for both of these states are shown in Figure 2b (before injection, no contrast) and Figure 2c (after injection, 6-fold pattern) and represent, qualitatively, the expected SAXS after successful skyrmion injection.

Previous theoretical works have studied current injection in smaller stepped geometries of skyrmion-containing systems using micromagnetics.^39,40^ To draw an analogy to these works, we also explicitly simulated a 50 ns STT current pulse (7.5 × 10^11^ A m^–2^) applied to the initial state shown in Figure 2d. The resulting magnetization profile after this current injection is shown in Figure 2f. As demonstrated previously,^39^ this simulated injection process results in skyrmion tubes that are bent near the thickness step in order to terminate on the vertical edge (also present to a lesser extent in Figure 2e, see expanded images in SI(38)). This is thought to arise from edge potentials, surface pinning, and an energetic preference to terminate at a surface, rather than form a Bloch point.^41,42^ This behavior is also very similar to the micromagnetics presented by Koshibae et al., who found that surface pinning restricts the ability of skyrmions near the surface to move past a thickness step while allowing the bulk of the skyrmion past, leading to bent states similar to those in our micromagnetics.^40^

As demonstrated by Figure 2f, the result of these considerations is a disordered skyrmion state in the thick region after current pulse. However, there are some important limitations of micromagnetics that restrict their direct comparison to real behavior, and hence require experimental study. First, in real samples the process of ion-thinning will result in a smoothly varying thickness profile rather than a step. This would serve to dampen the effects of surface-pinning, likely reducing the amount of disorder introduced into the lattice. Properly simulating such blurred profiles is more challenging in micromagnetics, and to allow a consistent comparison with previous theoretical works,^39,41,42^ a sharp step is maintained in our work. Next, the larger size of the real sample would likely allow more room for the injected skyrmion lattice to relax away from the thickness step, again likely reducing the disorder. Finally, and most importantly, micromagnetics is inherently a zero temperature technique. This has two important consequences. First, real systems will have thermal energy that may allow the skyrmions to rearrange into a less disordered and lower energy lattice post-injection. Second, micromagnetics usually fail to properly renucleate skyrmions in bulk DMI systems such as FeGe, due to the importance of thermal fluctuations. Hence, in Figure 2f, skyrmions do not reform in the thin region after the simulated current pulse (so skyrmion injection would be a one-time event), in contrast to a real system where they would reform as the equilibrium *B*–*T* conditions are satisfied. As a result of these limitations, while it provides a useful guide for the behavior of skyrmions in confined geometries, it is inadequate for completely understanding real systems. We therefore now turn to our experimental investigation of a thickness-step skyrmion injection device to provide additional insight on the real behavior of such systems.

To systematically observe the current effects as a function of magnetic phase, we performed a magnetic field sweep from 0–130 mT in steps of 10 mT after ZFC to 255 K. At each field, we applied 5 times 100 ms, 4 × 10^8^ A m^–2^ pulses in the injection direction, first collecting an initial scattering pattern, then measuring after each subsequent pulse. As expected, at low fields we observe a helical phase (see SI(38)) with little preference of direction due to the weak magnetocrystalline anisotropy present in FeGe at these temperatures.^43−45^ With each pulse we observe a rotation of the helical wavevector indicating the coupling between the magnetic moments and conduction electrons.^46^ At higher fields, we see skyrmion injection (Figure 3). At 70 mT (indicated by the star in Figure 2a), we start with no magnetic contrast in the scattering pattern (Figure 3a). From the phase diagram (Figure 2a), we expect a skyrmion state within the thinner region, consistent with Figure 2d. However, when we then apply an injection pulse, we see no differences in the scattering pattern of the thick region until after the fourth pulse, at which point 6-fold diffraction peaks are observed, indicative of a skyrmion lattice. On reversing the current direction, we start with skyrmion diffraction peaks (Figure 3f) that grow disordered (g, h) and then vanish with further pulses (Figure 3i). These results demonstrate reversible injection and ejection of the skyrmion lattice across the thickness step.

**Figure 3:**
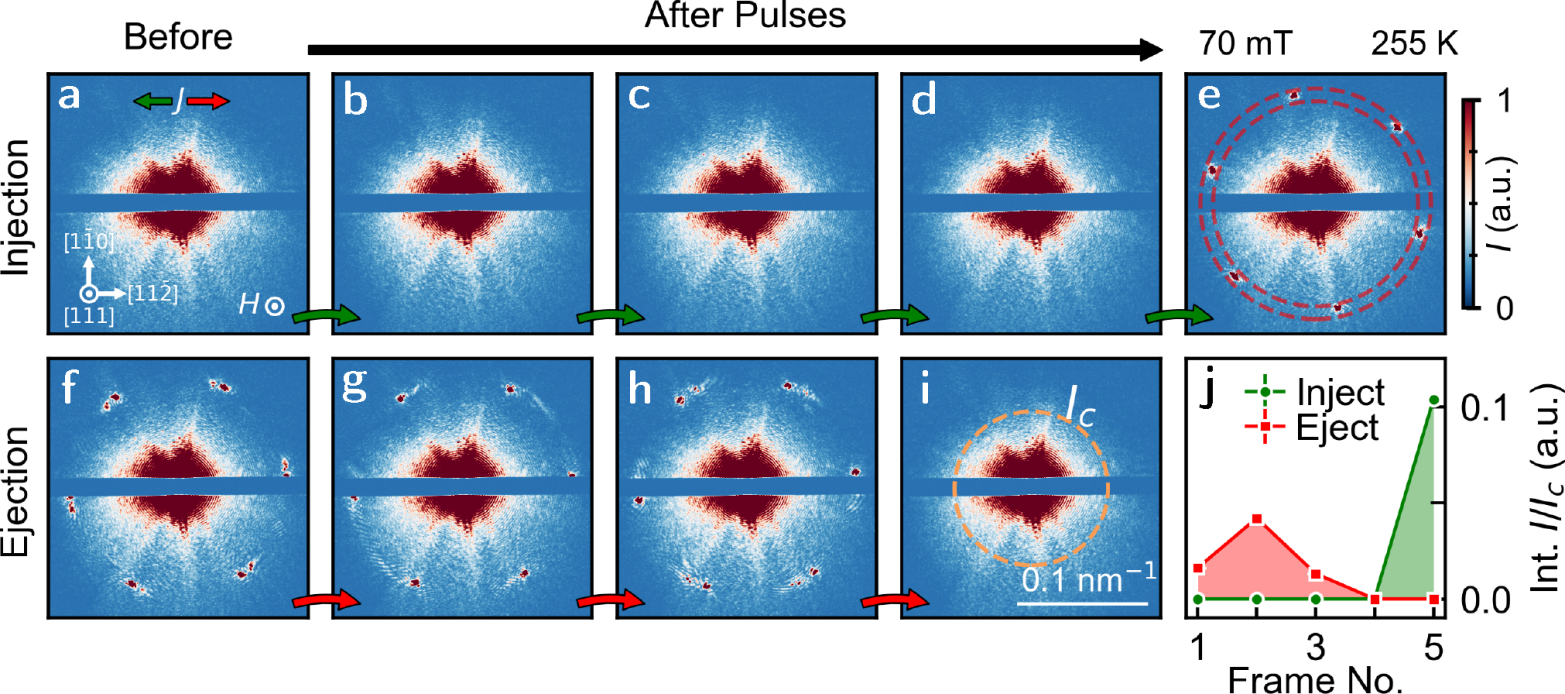
(a–i) Sequential scattering images after 0.1 s pulses with a current density of 4 × 10^8^A m^–2^ at 70 mT after ZFC to 255 K. Green arrows indicate an injection pulse (I_+_), and red arrows indicate an ejection pulse (I_–_). The corresponding directions of conventional current flow are indicated by the schematic in (a), i.e., green arrow (*I*_+_) refers to current flow from thick to thin. Schematic of crystallographic directions and magnetic field direction shown in (a). (j) Integrated intensity (normalized to charge scatter (orange circle in (i)) in the red circle shown in (e) vs frame for injection (green circles) and ejection (red squares).

Before continuing, it is worth discussing some characteristics of the diffraction patterns in Figure 3. First, injection of the skyrmion lattice occurs only after multiple current pulses. Since our knowledge of skyrmion injection comes from the observation of diffraction peaks before and after a current pulse, we are sensitive only to a macroscopic and long-range ordered skyrmion lattice rather than single skyrmions. Similarly, due to the experimental geometry, we are limited to a specific field-of-view within the device. This could explain the absence of scattering in the first three pulses of Figure 3a–d since skyrmions may be transported across the thickness barrier, but not be within our field of view. Notably, the transition from no magnetic contrast to 6-fold diffraction peaks confirms skyrmion injection, i.e., a transition from a zero skyrmion state into a finite skyrmion state, rather than the ordering of a disordered metastable phase in which we would expect a diffuse ring or disk of intensity condensing into 6-fold satellites. Second, in contrast to the simulated case, the skyrmions are significantly more well-ordered. As described above, the differences are likely explained by the thickness step approximating a smoothly varying function rather than an ideal discontinuity, leading to less pinning at the interface and subsequently less disorder. To fully quantify the extent of disorder and the effects of surface pinning due to the step, real-space imaging techniques such as X-ray holography and tomography will be required, beyond the scope of this study.

Examining these scattering patterns in further detail, we calculate the integrated intensity (indicated by the ring in Figure 3e) before and after the five injection pulses to characterize the optimal magnetic field and temperature conditions. Figure 4a shows the integrated intensity normalized to charge scattering (*I*_c_, integrated intensity within the circle in Figure 3i) before and after the five injection pulses for a field scan between 0 and 130 mT. At low fields (between 0 and 50 mT), the integrated intensities are, within error, identical, indicating that the current effects do nothing to the volume fraction of the helical phase. Between 50 and 80 mT, we observe significant differences. After injection, the scattered intensity increases due to the presence of a skyrmion lattice within the aperture. At 80 mT, the existence of an equilibrium skyrmion lattice limits skyrmion injection, and now the application of a current pulse changes the scattered intensity by 6–12%. This represents a change in skyrmion volume fraction within the field-of-view. COMSOL simulations of this geometry indicate a maximal change in temperature caused by Joule heating of 4 mK during the current application (Figure 4d, more details in SI(38)). Therefore, these changes in scattering patterns are unlikely to be caused by sample heating, and we interpret the 80 mT data as a change in the observed skyrmion lattice volume-fraction as the skyrmions move through the aperture. More clearly, the difference in the integrated intensities before and after injection is shown in Figure 4b, demonstrating the ideal injection fields at 255 K.

**Figure 4:**
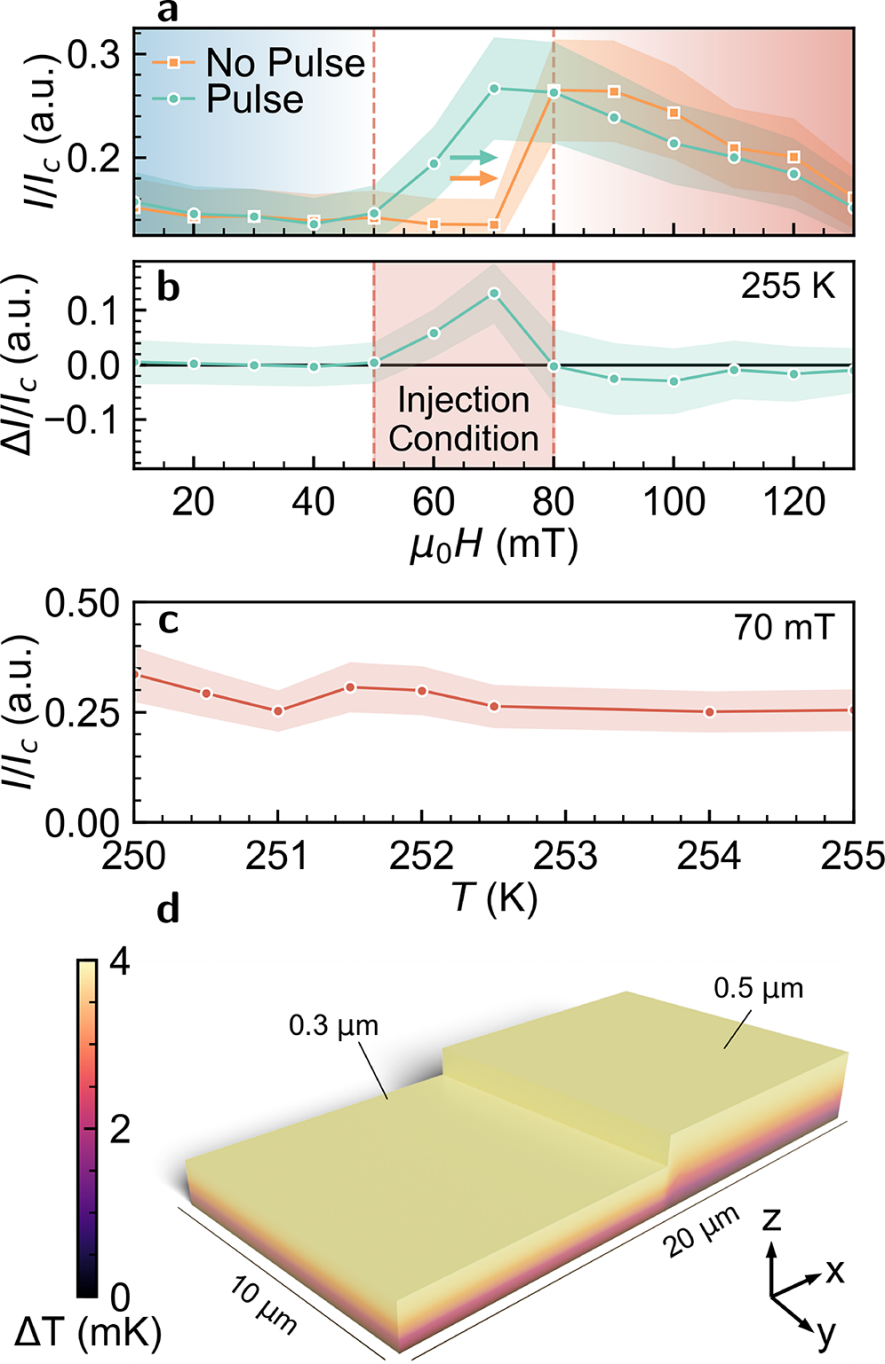
(a) Integrated intensity of scattering patterns during the field sweep at 255 K after ZFC from 300 K before and after injection. Arrows refer to the intensity captured on an increasing field for both data sets. (b) Difference in normalized intensity in (a) indicating injection between 50–70 mT. (c) Integrated intensity after injection at 70 mT as a function of temperature. Error bars in (a)–(c) are based off the standard error in the postinjection intensity calculated from Figure 5b. (d) Simulated Joule heating indicating a maximal temperature change of 4 mK. Z-height was scaled by 5 times for easier visual inspection.

Since 70 mT shows the maximum number of injected skyrmions, we perform a small temperature sweep from 250–255 K and integrate the intensity after a 1s 4 × 10^8^ A m^–2^ pulse to guarantee skyrmion injection (Figure 4c). We find that over the entire temperature range we can inject skyrmions into the thicker region with no significant correlation in the number of injected skyrmions to the temperature. Additionally, at 255 K, a fit of the skyrmion intensity over time indicates that the lifetime of the injected metastable state is approximately 18.5 h (see SI(38)). This highlights the applicability of the device as a skyrmion generation method over a range of temperatures, which can also likely be extended by producing a device with a larger difference in thickness between the two regions, thereby increasing the skyrmion equilibrium regime separation.

Finally, we characterize the electrical operation of the device and discuss some potential applications. Figure 5a,b demonstrates a series of consecutive injection and ejection pulses as a function of time. The normalized integrated intensity is then plotted in Figure 5b and demonstrates variation in the number of injected skyrmions. As discussed previously, the limited field-of-view does not allow an accurate measure of the number of injected skyrmions. Nevertheless we observe consistent injection and ejection over a repeated cycle of 10 alternating pulses. The consistency of ejection is another indicator that heating is not the primary mechanism of skyrmion nucleation, since heating is independent of current polarity, and we would therefore expect the skyrmions to be maintained throughout the pulse sequence.

**Figure 5:**
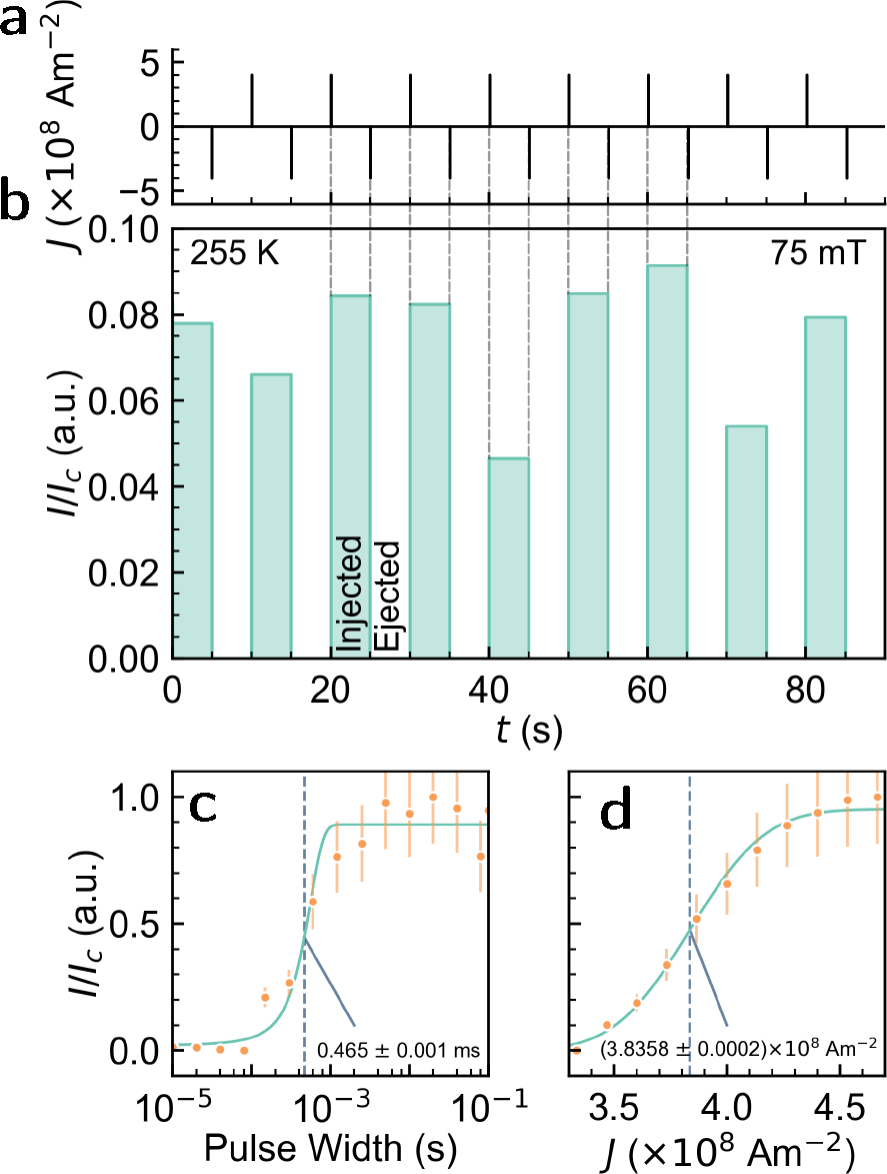
Sequential 100 ms current pulses of ±4 × 10^8^ A m^–2^ were applied alternating between negative and positive polarity. (a) Schematic of the pulse sequence applied (b) Normalized skyrmion intensity (*I*/*I*_*c*_) measured between each subsequent current pulse. (c) Normalized injected skyrmion intensity (relative to the maximum intensity) measured for varying pulse width at a current density of 3 × 10^8^ A m^–2^. (d) Intensity vs current density at a fixed pulse width of 0.1 s. Lines are guides to the eye and are fitted using , where *x*_0_ is taken as the injection threshold. Error bars indicate the relative error based on the standard error of the intensity post injection in (b), accounting for random fluctuations in the number of skyrmions injected.

Repeating the sequence of pulses indicated above, we fix the current density to ±4 × 10^8^ A m^–2^ and vary the pulse width between 10 μs and 100 ms. Integrated intensities are shown in Figure 5c, which indicate a threshold pulse width of 0.465 ms at a current density of ±4 × 10^8^ A m^–2^. Fixing the pulse width to 100 ms and varying the current density indicates a similar trend (Figure 5d). These electrical characteristics indicate potential applicability, where injection and the number of injected skyrmions can be chosen by a current density greater than 3 × 10^8^ A m^–2^ and subsequently moved with smaller current densities on the order of 1 × 10^6^ A m^–2^.^47^ Such characteristics are likely to be favorable in reservoir and neuromorphic computing regimes in which a write pulse can be used to initialize the skyrmion reservoir before subsequent pulses are used as an input.^48,49^ By then applying a larger ejection current the reservoir can be wiped and reinitialized providing complete electrical operation in constant *B*–*T* conditions. As previously discussed, the results of this work relate to the collective injection of numerous skyrmions across a thickness step. Within our 4.5 μm aperture, there would be approximately 1000 skyrmions, and hence, we are seeing several hundred skyrmions being injected/ejected. For applications that require single skyrmions, our technique may be extended following work on nanostructures similar to that of Birch et al.^35^ by using a stepped nanowire. However, more work on the dynamics of single skyrmions in confined geometries will be required to realize this. Moreover, understanding the extent of the metastability of injected skyrmions will provide further detail into the applicability of this in devices that require stable or fading memory. For this, we suggest transport measurements which can measure the instantaneous formation of skyrmions through their THE and thus characterize their decay over a prolonged lifetime.

In summary, utilizing the three-dimensional properties of skyrmions and their host materials, we have demonstrated current induced injection and ejection of skyrmions across a thickness step and into a region of metastability in a 300 nm/500 nm thick FeGe lamella. This injection is achieved by a low current density of 4 × 10^8^ A m^–2^, viable for applications. We further demonstrate the optimal conditions and electrical threshold characteristics of the injection mechanism and aim to point the way toward the development of a practical application for the mechanism within skyrmion logic and memory devices.

## Supporting Information Available

A number of movies (GIF) of simulated injections/ejections for a number of current densities and thickness ratios (ZIP)
Details of the sample preparation, further discussion of micromagnetic and physical property simulations, alongside potential device applications. Descriptions of the supporting movies provided in the zip file (PDF)
