# Supplementary figures and images for: Demonstration of Controlled Skyrmion Injection Across a Thickness Step
Source: Nano Lett. 2024 May 23;24(22):6813–20. doi: 10.1021/acs.nanolett.4c01605 (PMC11157652; doi:10.1021/acs.nanolett.4c01605)

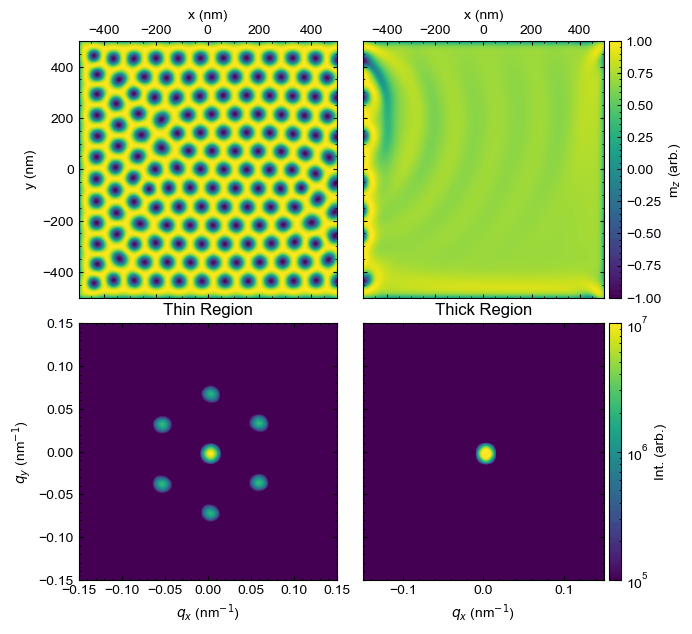

Supplement: Supplementary file 1 — nl4c01605_si_001.zip [file nl4c01605_si_001.zip › SupplementaryMovies/MovieS12.gif]

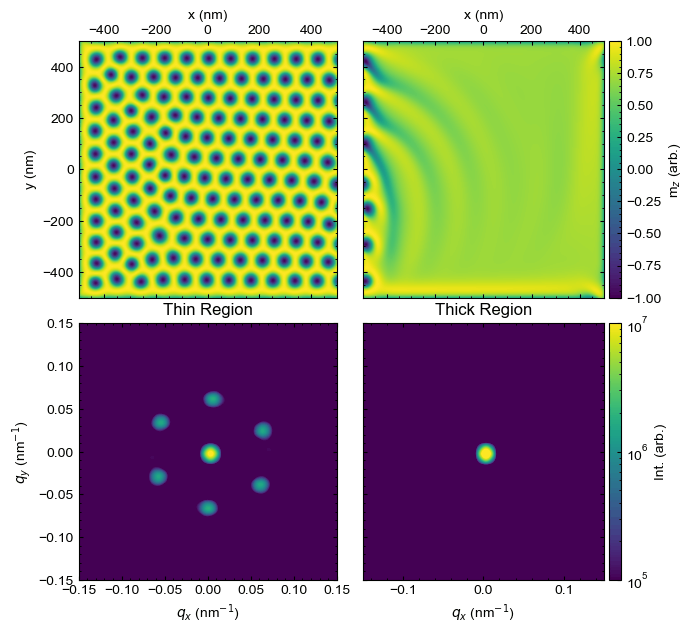

Supplement: Supplementary file 1 — nl4c01605_si_001.zip [file nl4c01605_si_001.zip › SupplementaryMovies/MovieS13.gif]

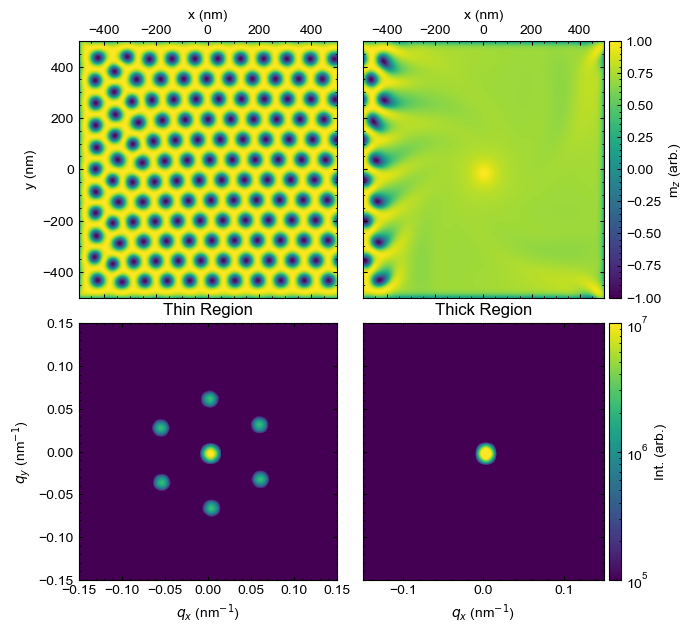

Supplement: Supplementary file 1 — nl4c01605_si_001.zip [file nl4c01605_si_001.zip › SupplementaryMovies/MovieS11.gif]

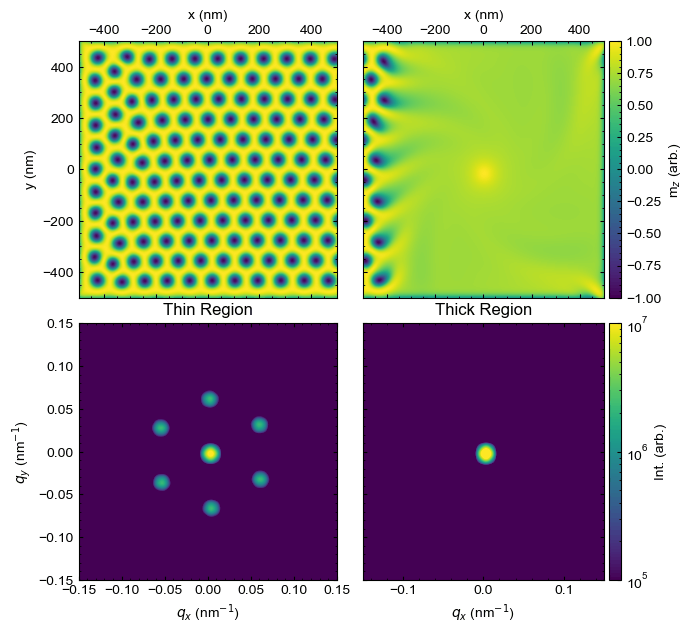

Supplement: Supplementary file 1 — nl4c01605_si_001.zip [file nl4c01605_si_001.zip › SupplementaryMovies/MovieS10.gif]

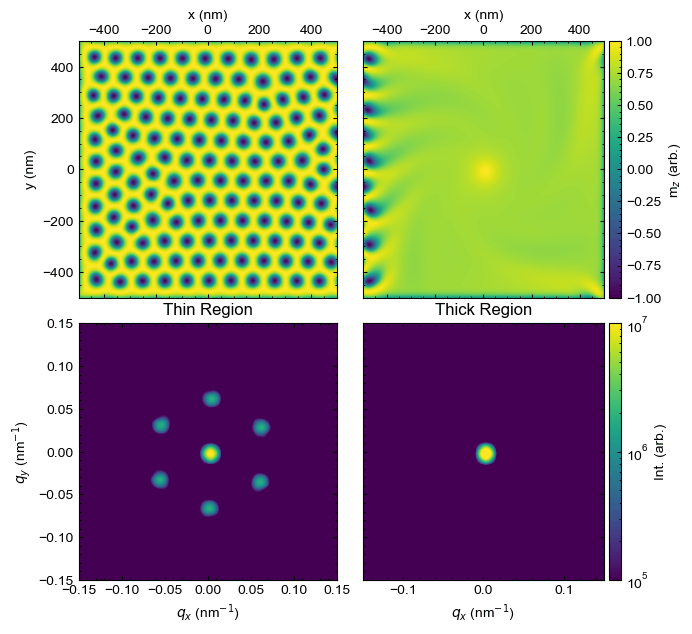

Supplement: Supplementary file 1 — nl4c01605_si_001.zip [file nl4c01605_si_001.zip › SupplementaryMovies/MovieS14.gif]

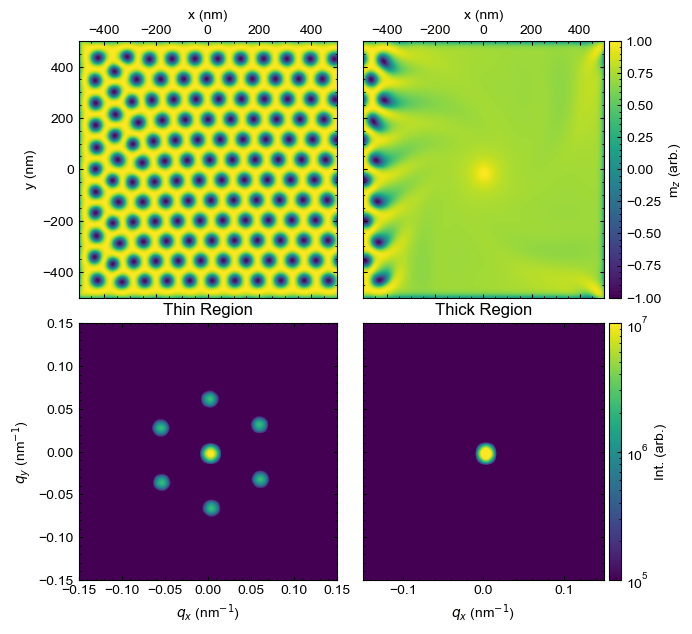

Supplement: Supplementary file 1 — nl4c01605_si_001.zip [file nl4c01605_si_001.zip › SupplementaryMovies/MovieS9.gif]

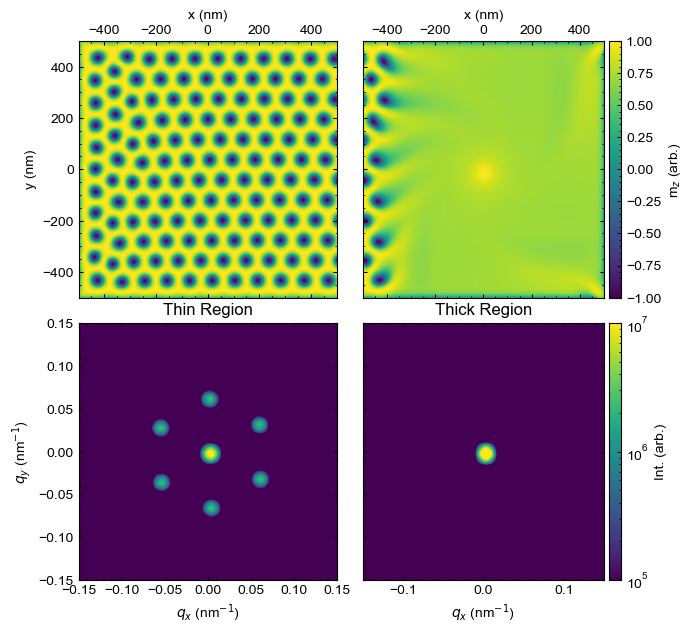

Supplement: Supplementary file 1 — nl4c01605_si_001.zip [file nl4c01605_si_001.zip › SupplementaryMovies/MovieS8.gif]

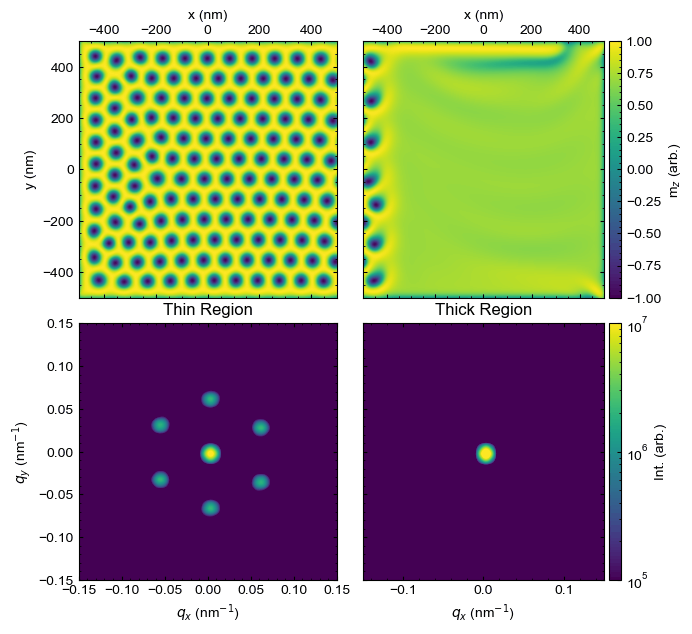

Supplement: Supplementary file 1 — nl4c01605_si_001.zip [file nl4c01605_si_001.zip › SupplementaryMovies/MovieS15.gif]

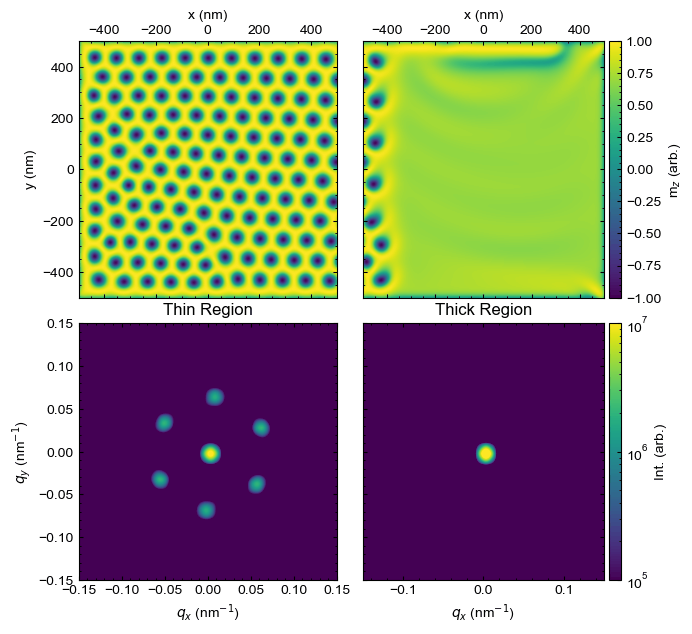

Supplement: Supplementary file 1 — nl4c01605_si_001.zip [file nl4c01605_si_001.zip › SupplementaryMovies/MovieS17.gif]

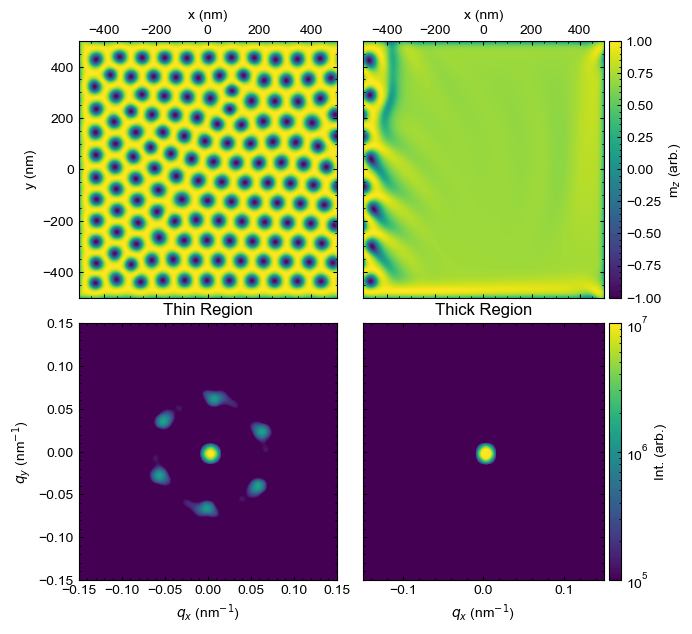

Supplement: Supplementary file 1 — nl4c01605_si_001.zip [file nl4c01605_si_001.zip › SupplementaryMovies/MovieS16.gif]

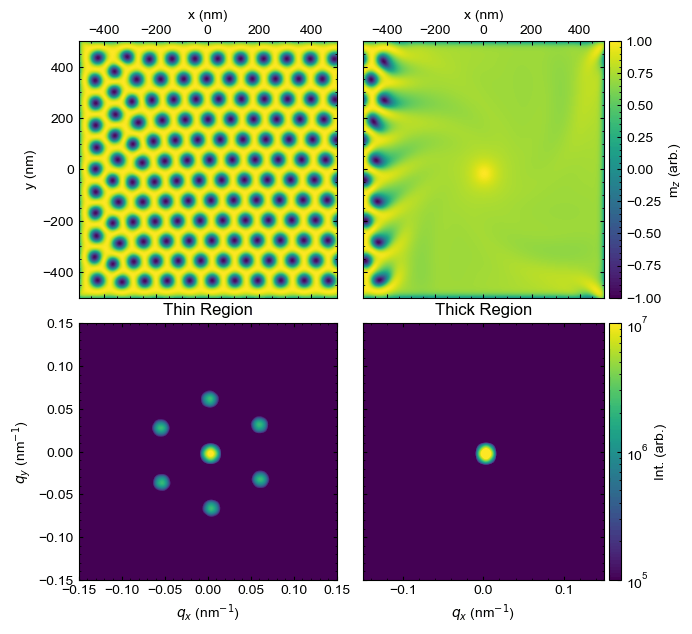

Supplement: Supplementary file 1 — nl4c01605_si_001.zip [file nl4c01605_si_001.zip › SupplementaryMovies/MovieS6.gif]

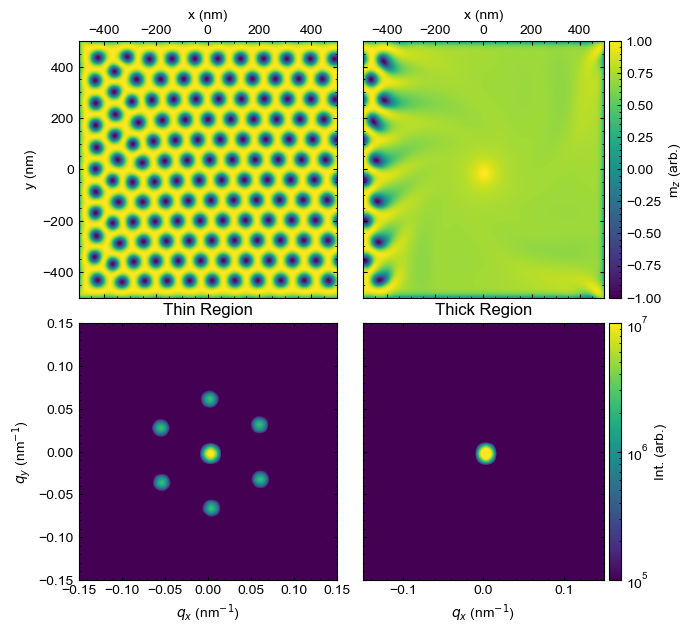

Supplement: Supplementary file 1 — nl4c01605_si_001.zip [file nl4c01605_si_001.zip › SupplementaryMovies/MovieS7.gif]

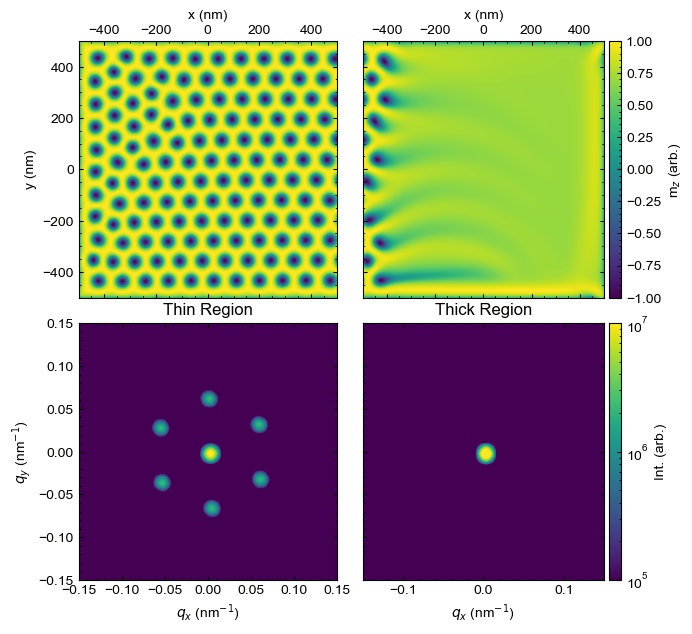

Supplement: Supplementary file 1 — nl4c01605_si_001.zip [file nl4c01605_si_001.zip › SupplementaryMovies/MovieS18.gif]

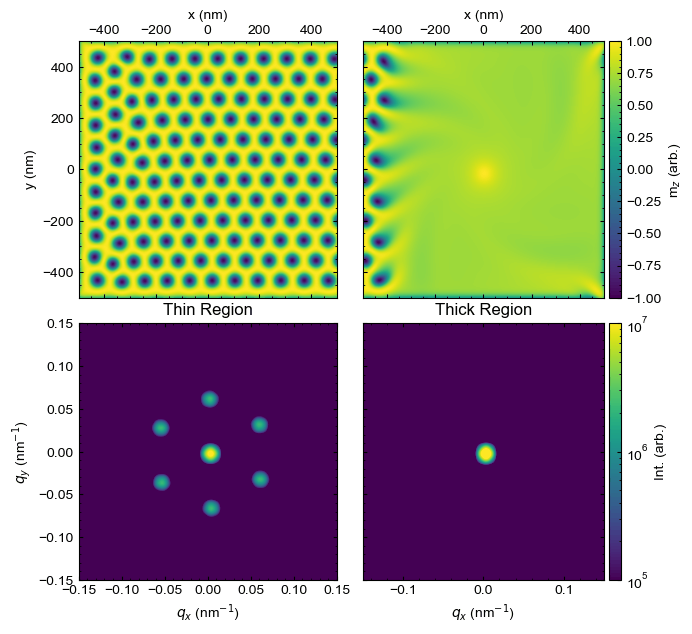

Supplement: Supplementary file 1 — nl4c01605_si_001.zip [file nl4c01605_si_001.zip › SupplementaryMovies/MovieS5.gif]

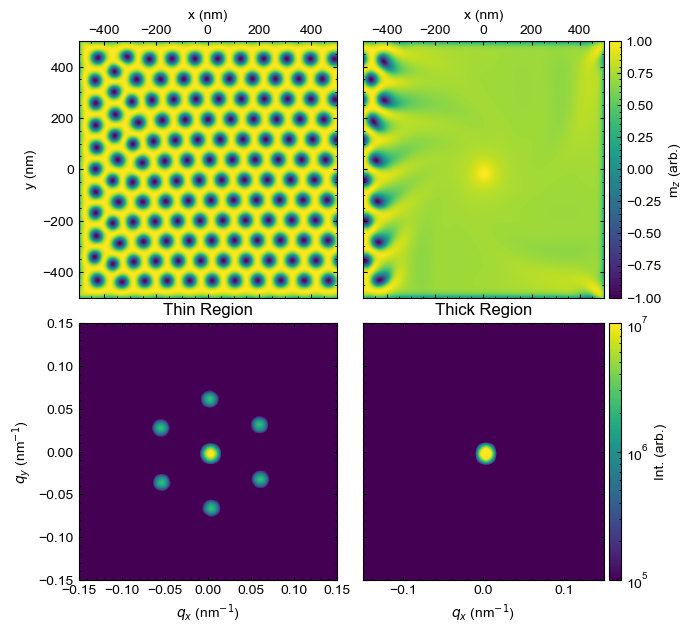

Supplement: Supplementary file 1 — nl4c01605_si_001.zip [file nl4c01605_si_001.zip › SupplementaryMovies/MovieS4.gif]

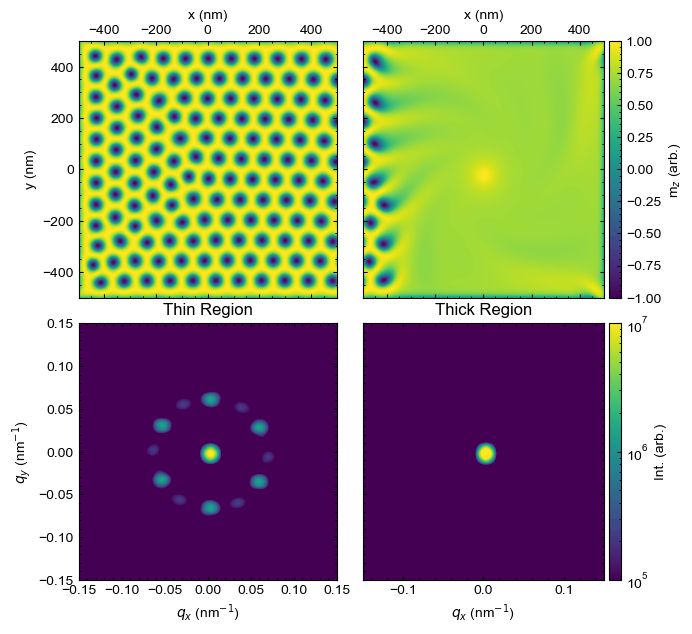

Supplement: Supplementary file 1 — nl4c01605_si_001.zip [file nl4c01605_si_001.zip › SupplementaryMovies/MovieS19.gif]

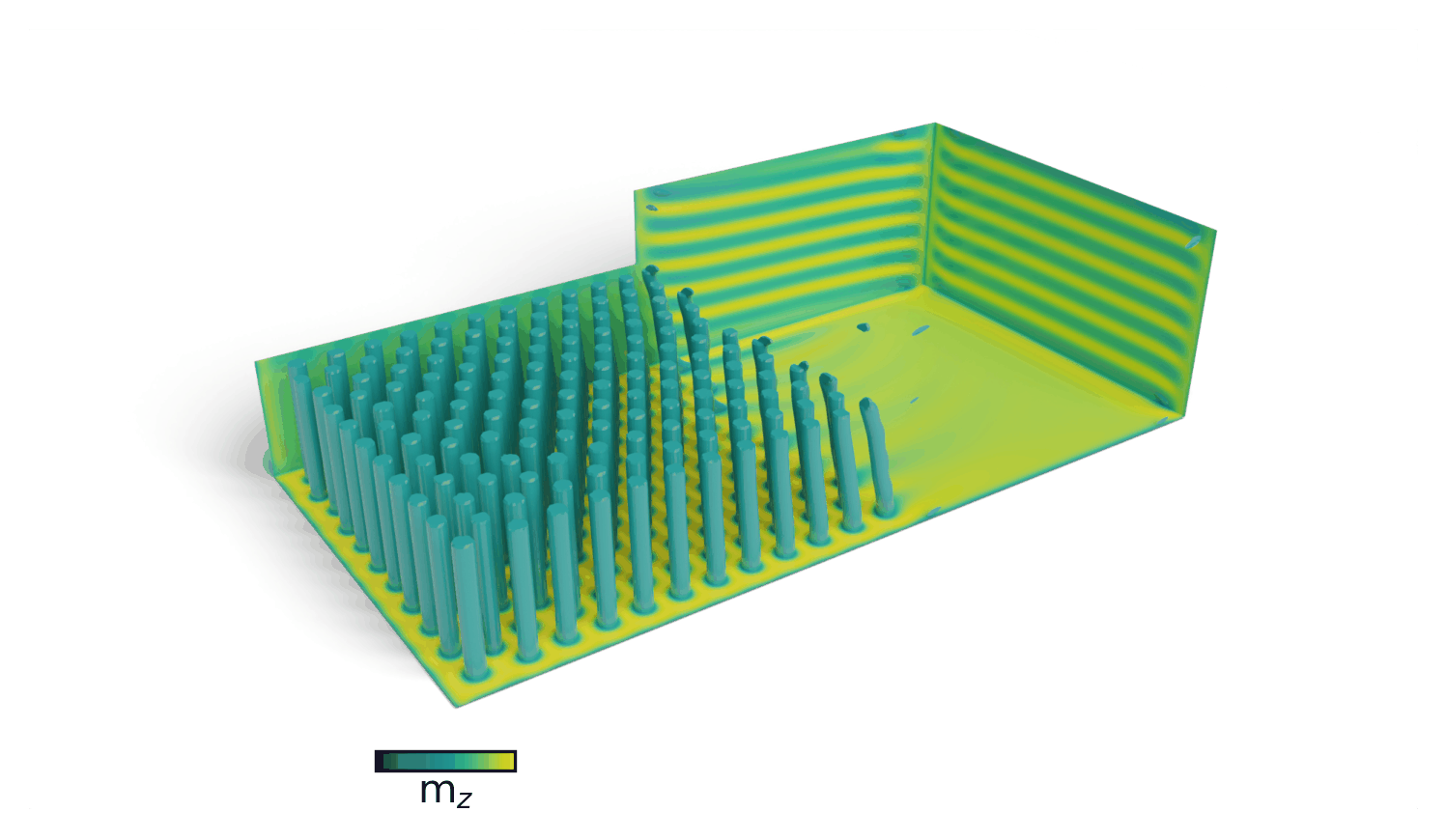

Supplement: Supplementary file 1 — nl4c01605_si_001.zip [file nl4c01605_si_001.zip › SupplementaryMovies/MovieS1.gif]

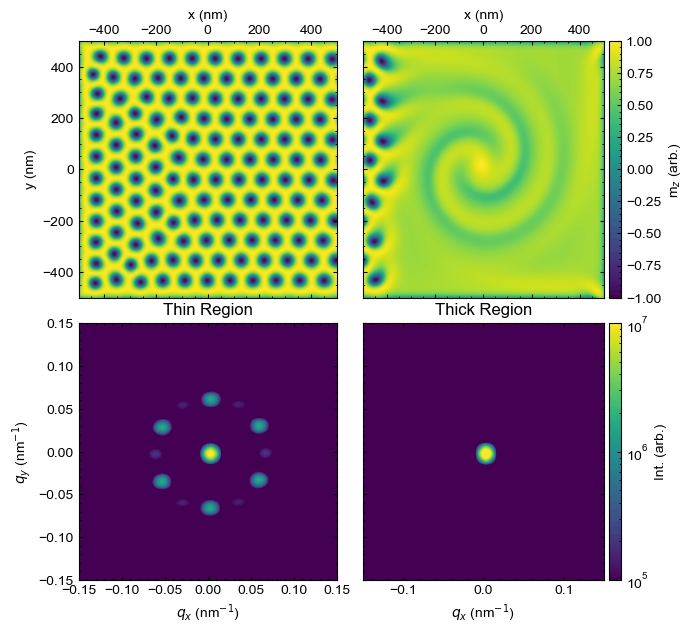

Supplement: Supplementary file 1 — nl4c01605_si_001.zip [file nl4c01605_si_001.zip › SupplementaryMovies/MovieS20.gif]

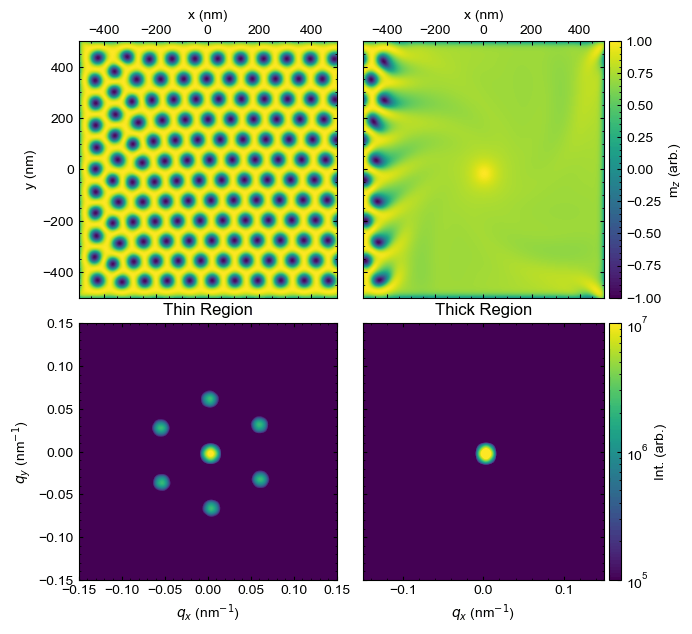

Supplement: Supplementary file 1 — nl4c01605_si_001.zip [file nl4c01605_si_001.zip › SupplementaryMovies/MovieS3.gif]

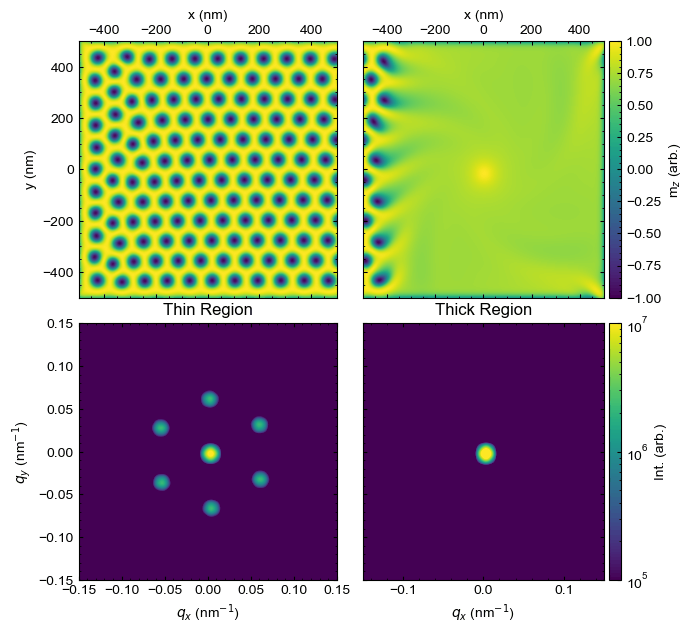

Supplement: Supplementary file 1 — nl4c01605_si_001.zip [file nl4c01605_si_001.zip › SupplementaryMovies/MovieS2.gif]
