# Supplementary material for: Demonstration of Controlled Skyrmion Injection Across a Thickness Step
Source: Nano Lett. 2024 May 23;24(22):6813–20. doi: 10.1021/acs.nanolett.4c01605 (PMC11157652; doi:10.1021/acs.nanolett.4c01605)
Supplement: Supplementary file 2 — nl4c01605_si_003.pdf [file nl4c01605_si_003.pdf]

Demonstration of controlled skyrmion injection across a thickness step: Supplementary Information

Matthew T. Littlehales,[*,†,‡,△] Samuel H. Moody,[†,¶] Luke A. Turnbull,[†,§] Benjamin M. Huddart,[†,‖] Ben A. Brereton,[†] Geetha Balakrishnan,[⊥] Raymond Fan,[#] Paul Steadman,[#] Peter D. Hatton,[*,†] and Murray N. Wilson[†,@,△]

†*Durham University, Department of Physics, South Road, Durham, DH1 3LE, United Kingdom*

‡*ISIS Neutron and Muon Source, Rutherford Appleton Laboratory, Didcot, OX11 0QX, United Kingdom*

¶*Laboratory for Neutron Scattering and Imaging, Paul Scherrer Institute, Villigen, CH-5232, Switzerland*

§*Max Planck Institute for Chemical Physics of Solids, Noethnitzer Str. 40, 01187 Dresden, Germany*

‖*Department of Physics, Clarendon Laboratory, University of Oxford, Parks Road, Oxford, OX1 3PU, United Kingdom*

⊥*University of Warwick, Department of Physics, Coventry, CV4 7AL, United Kingdom*

#*Diamond Light Source, Didcot, OX11 0DE, United Kingdom*

@*Memorial University of Newfoundland, Department of Physics and Physical Oceanography, St John's, Newfoundland, A1B 3X7, Canada*

△*These authors contributed equally to this work*

E-mail: matthew.t.littlehales@durham.ac.uk; p.d.hatton@durham.ac.uk

Device fabrication

The bulk, $1.5 \times 1.5 \times 1.5\,\mathrm{mm}^3$ single crystals of FeGe were grown via the chemical vapor transport method using 2 g of prepared FeGe powder and 2 mg/cm3 of iodine transport agent with the source maintained at 250 °C and a 50 °C temperature gradient across the length of the furnace for 1-2 weeks. A lamella of FeGe was lifted from a single crystal using an FEI Helios Nanolab Mk2 focused ion beam instrument with the $[1\,1\,1]$ direction aligned out of the plane, and $[1\,1\,\bar{2}]$ and $[1\,\bar{1}\,0]$ directions along the two perpendicular edges of the lamella. This alignment was determined using electron diffraction with 200 kV electrons in a JEOL 2100F FEG transmission electron microscope on a $1 \times 5\,\mu\mathrm{m}$ portion of the lamella. The sample was then shaped into the geometry defined within the main text, a plate with thicknesses of 300 nm and 500 nm separated by a step-like feature. The device was then placed over a 4.5 µm aperture milled through a Au coated Si_3N_4 membrane which had Au electrodes patterned over the top. The device was attached to the membrane using Pt deposition such that the edges of the lamella in direct physical contact with the Au electrodes.

Fig. 1 shows images of the samples measured and corresponding electron diffraction images confirming the sample alignment.

Small angle x-ray scattering

The Si_3N_4 chip holding the injector device was mounted onto a copper sample holder using silver paint and enamel coated copper wires were attached to the membrane electrodes also using silver paint. Care was taken to avoid shorting the membrane during the mounting process. The enamel wires were then converted into a coaxial cable which fed through the instrument into a Keithley 3390 50 MHz Arbitrary Waveform Generator. This power supply was then used to send single square wave pulses with pulse lengths between 10 µs and 10 s, and constant voltage amplitudes up to 350 mV, which were verified to pass through the sample using a Tekronix DPO3014 oscilloscope.

The sample holder itself was mounted on a copper cold finger inside the RASOR end

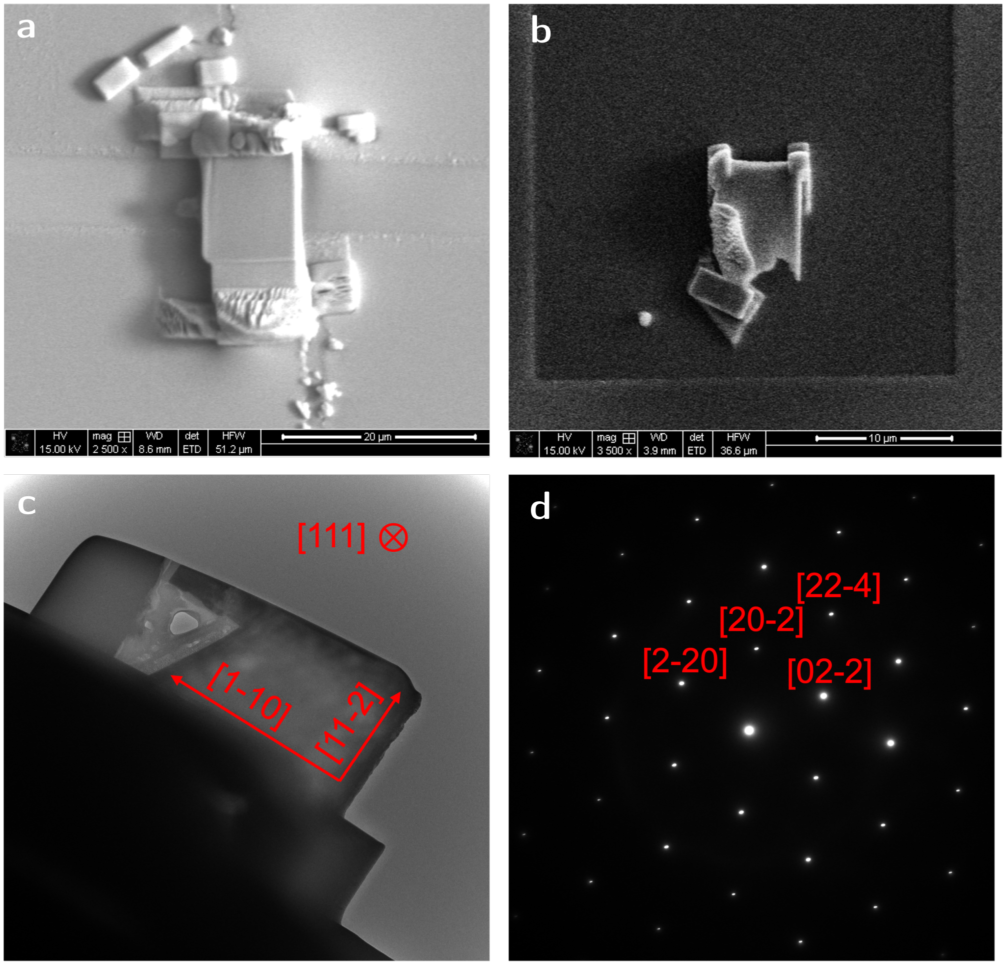

Figure 1: Scanning electron microscopy image (SEM) of injector sample (a) and thin region taken from same sample (b). Transmission electron microscopy image (c) and diffraction pattern (d) confirming alignment.

station of the I10 beamline at Diamond Light Source, and cooled using liquid nitrogen. Magnetic fields between 0 and 160 mT were applied using a four pillar permanent magnet array (two pillars behind and in front of sample to avoid blocking incident beam) which was moved relative to the sample to change the magnetic field. Using this instrument, we performed SAXS measurements using circularly polarized x-rays at the Fe L_3 absorption edge. Scattered x-rays were detected using a 2048 $\times$ 2048 pixel CCD (pixel width 13.5 µm) mounted 138 mm from the sample, for a Q-resolution of 3.5×10^{-4} nm^{-1}. A horizontal beam stop was used to avoid damaging the detector with the intense directly transmitted beam.

Injection Field Scans

As mentioned in the main text, we performed a magnetic field scan after zero field cooling to 255 K and performed 5x injection and ejection pulses. Fig. 2 summarises this field sweep at 10 mT, 70 mT and 80 mT. At 10 mT we observed rotations of the wavevectors in the helical phase caused by spin transfer torque coupling. At 70 mT we initially observe no magnetic contrast indicative of a conical or field polarised phase, then after 5x pulses we observed a six fold pattern indicative of a skyrmion lattice. Then after 3x ejection pulses the contrast disappears indicative of a loss of the skyrmion lattice from the observable aperture. Finally, at 80 mT, we observe skyrmions in the first image, indicative that we are within the equilibrium skyrmion pocket.

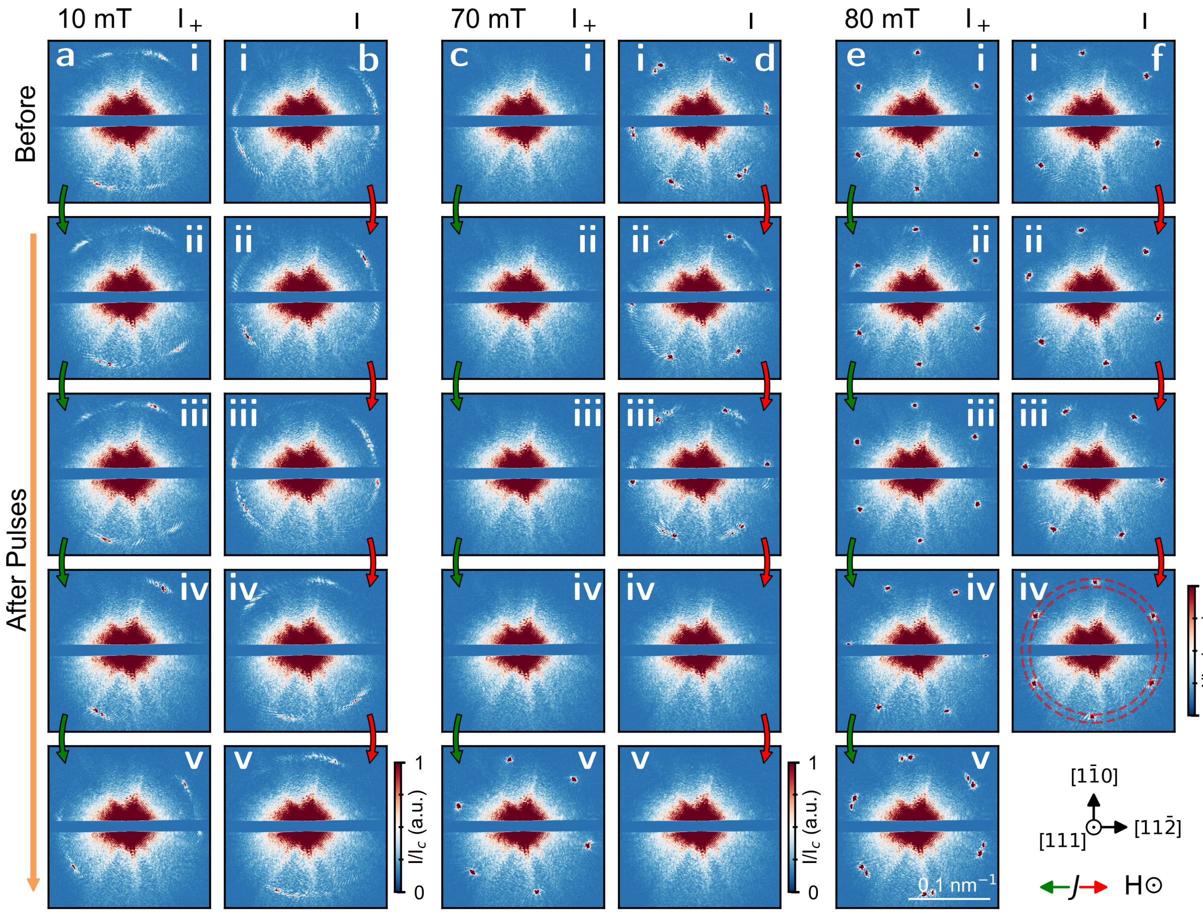

Figure 2: Magnetic field scan at 255 K after zero-field cooling with 5x current pulses at each field. Injection and ejection pulses shown for 10 mT (a, b(i-v)), 70 mT (c, d(i-v)), and 80 mT (e, f(i-v)).

Micromagnetic Simulations

Simulations of the skyrmion injection mechanism were performed using the Ubermag micromagnetics package[1,2] utilising the OOMMF micromagnetic calculator.[3] To simulate the device geometry, we use the simplest model interactions required for skyrmion formation in FeGe with $P2_13$ space group (direct exchange, Dzyaloshinskii Moriya interaction (DMI), Zeeman interaction), and obtain the energy functional as:[4]

$$E = \int_V dV(-A\mathbf{m} \cdot \nabla^2 \mathbf{m} + D\mathbf{m} \cdot (\nabla \times \mathbf{m}) + \mu_0 M_S \mathbf{m} \cdot \mathbf{H}) \tag{1}$$

where $\mathbf{m}$ is the normalised magnetisation field, $A = 8.78\,\mathrm{pJ\,m^{-1}}$ is the exchange strength, $D = 1.58\,\mathrm{mJ\,m^{-2}}$ is the DMI strength, $M_S = 384\,\mathrm{kA\,m^{-1}}$ is the saturation magnetisation, and $\mathbf{H}$ is the magnetic field vector. We use standard previously reported FeGe parameters[5] which yield an exchange length of $l_{\mathrm{ex}} = 9.73\,\mathrm{nm}$. Subsequently, we use $5{\times}5{\times}5\,\mathrm{nm}$ cells such that the cell size is smaller than the exchange interaction length to build a geometry consisting of a $2\,\mu\mathrm{m}$ x $1\,\mu\mathrm{m}$ slab with thicknesses of $300\,\mathrm{nm}$ and $500\,\mathrm{nm}$ interfaced at the center of the geometry. We seed an initial skyrmion state into the thin region by initialising a square grid of cylindrical magnetisation aligned with the -z direction, antiparallel to the surrounding background magnetisation which is then relaxed within a $210\,\mathrm{mT}$ magnetic field aligned along the positive z-direction using a conjugate gradient energy minimiser.

To drive the system with a spin-polarised current, we solve the Landau-Lifshiftz-Gilbert equation with the addition of a Zhang-Li spin-transfer torque term:[6]

$$\begin{aligned}\frac{\partial \mathbf{m}}{\partial t} = &-\frac{\gamma_0}{1+\alpha^2}\mathbf{m} \times \mathbf{H}_{\mathrm{eff}} \\ &-\frac{\gamma_0 \alpha}{1+\alpha^2}\mathbf{m} \times (\mathbf{m} \times \mathbf{H}_{\mathrm{eff}}) \\ &-\frac{1+\alpha\beta}{1+\alpha^2}\mathbf{m} \times (\mathbf{m} \times (\mathbf{u} \cdot \boldsymbol{\nabla})\mathbf{m}) \\ &-\frac{\beta-\alpha}{1+\alpha^2}\mathbf{m} \times (\mathbf{u} \cdot \boldsymbol{\nabla})\mathbf{m},\end{aligned} \tag{2}$$

where $\mathbf{m}$ is the normalised magnetisation field, γ is the gyromagnetic ratio, α is the Gilbert damping parameter chosen to be 0.066 for FeGe,[7] and β is the non-adiabatic spin transfer torque parameter, chosen to be 0.[8] Here $\mathbf{u}$ denotes a velocity vector which includes the spin current density through the relation:

$$\mathbf{u} = -\frac{Pg\mu_B}{2eM_S}\mathbf{J}, \tag{3}$$

where P is the spin polarisation, g the Landé g-factor, μ_B the Bohr magneton, e the positive unit charge, M_S the saturation magnetisation, $\mathbf{H}_{\text{eff}}$ the effective magnetic field, and $\mathbf{J}$ the current density. Differences in the magnetic field values used to stabilise the initial state and the lack of skyrmion regeneration post current pulse are likely to be explained by the zero temperature approximation used within the micromagnetic model. In the following results, the topological charge is quantified using a continuous approach and calculated within the Ubermag package using:

$$Q = \frac{1}{4\pi}\boldsymbol{n} \cdot \left(\frac{\partial \boldsymbol{n}}{\partial x} \times \frac{\partial \boldsymbol{n}}{\partial y}\right) \tag{4}$$

Fig. 3 demonstrates the simulation of the full injector with a zoomed in image of the slight bending of the skyrmion caused by the step edge.

Current Density Dependence

To quantify an approximate threshold current density for the injection of skyrmions across a 300 nm / 500 nm thickness barrier, we simulated a 50 ns current pulse with varying current density and track the topological charge within the z=0 plane. An example of this is demonstrated in Fig. 4. We then reduce the simulations into Fig. 4, where we plot the topological charge of the in each of the thin and thick regions for both injection and ejection. Notably, no significant changes are observed up to 3.32×10^{10} A m^{-2}, then at 1.54×10^{11} A m^{-2} we see a change in the topological charge of the thin region (Fig. 4(a)) over the course of the current pulse. After this there is significant injection of the skyrmions into the thicker region

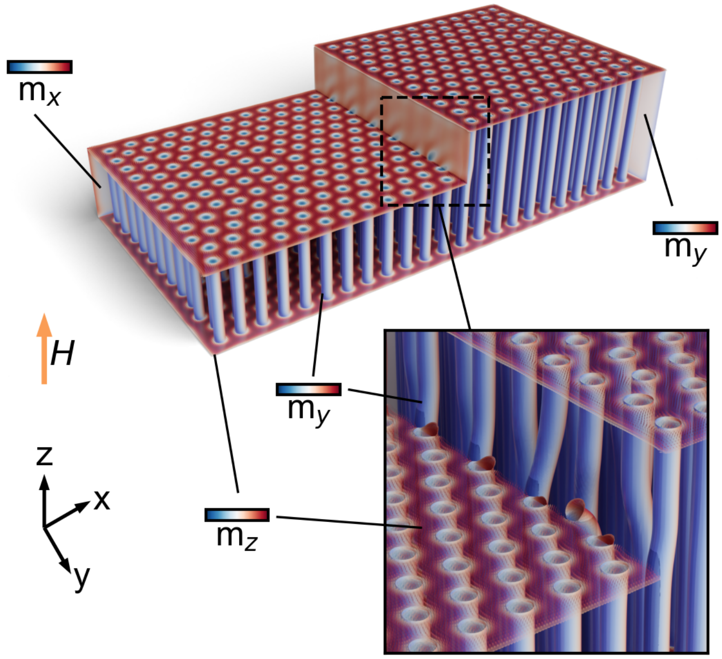

Figure 3: Micromagnetic simulation of skyrmions within full injector device. Inset shows zoomed in image of disorder induced by step.

of the device at $7.5 \times 10^{11}\,\mathrm{A\,m^{-2}}$ and at $3.32 \times 10^{12}\,\mathrm{A\,m^{-2}}$, the current density is enough to completely destroy the skyrmion lattice after they have been injected. Fig. 5 shows the topological charge versus time for both regions for injection (a,b) and ejection (c,d) and movie's for current each current density are also shown in Movies (S2-S11)

Device thickness dependence

The operating conditions of the device can be tailored into a specific temperature and field regime by varying the thicknesses of each section of the device. Since the surfaces and confinement play an important role in the dynamics of the spin texture, it is important to understand how the thickness differences affects the skyrmion injection. Consequently, we have performed multiple simulations with varying only the thickness ratio between the two regions of the device. By changing the thickness of only the thin region while maintaining a thickness of 500 nm in the thick region, we apply a 50 ns pulse with a current density of

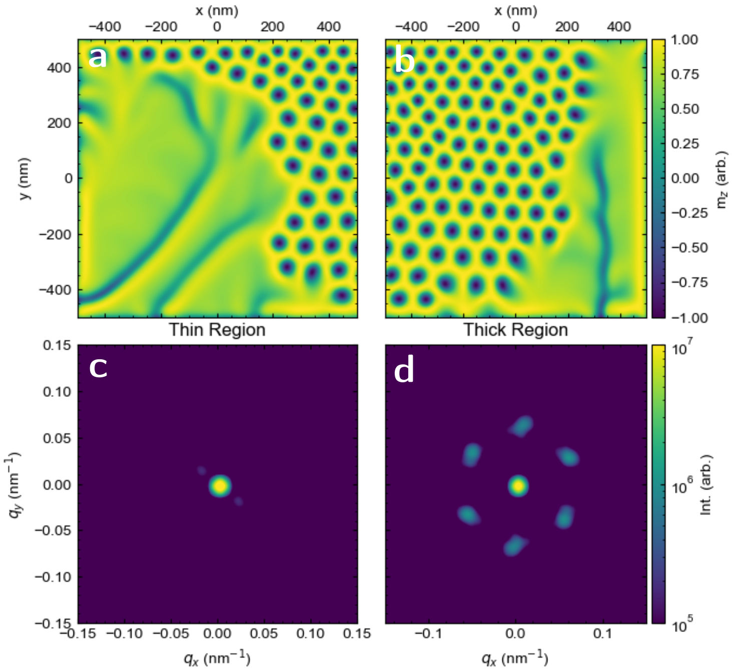

Figure 4: Micromagnetic simulation of injector device midway through current pulse for thin (a) and thick(b) regions, with corresponding Fourier transforms (c,d).

$7.5 \times 10^{11}\,\mathrm{A\,m^{-2}}$, the optimal current for injection from the previous current density series. As in the case of the current density dependence, we use the z=0 plane topological charge to characterise the effectiveness of the injection.

As shown in Fig. 6, the changes in thickness ratio do not render the skyrmion injection process impossible. However, as the ratio between the two thicknesses becomes large, injection becomes more difficult, likely due to the amount the skyrmion must deform in order to change it's length by a significant amount. Therefore, it is better to maintain a small difference in thickness to maintain a reasonably sized injection condition while still injecting a large number of skyrmions. Nevertheless, this is another tunable parameter.

Current Density and Joule heating simulations

The commercially available COMSOL Multiphysics (COMSOL Inc.) package was used to simulate the current density distribution throughout the sample geometry alongside Joule

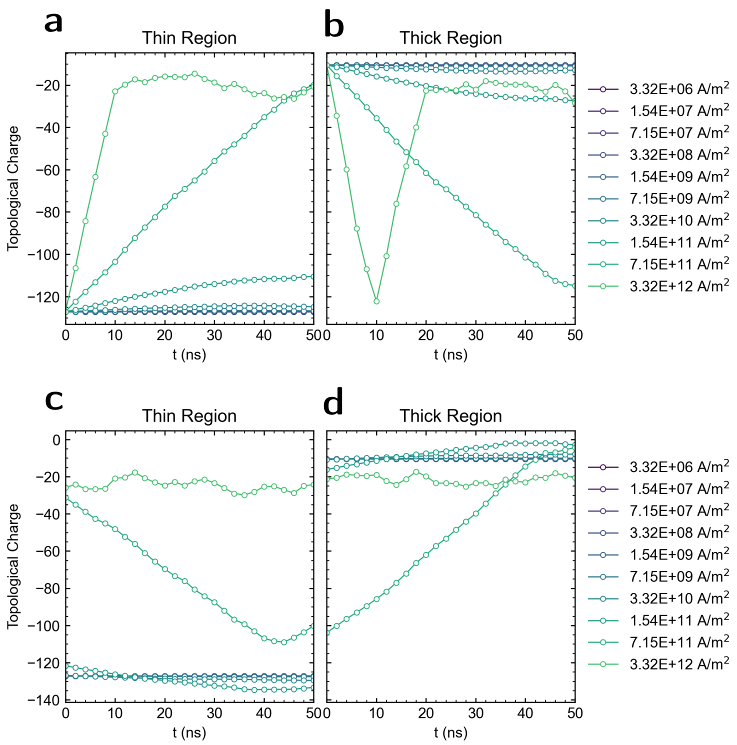

Figure 5: Z = 0 topological charge within each of section of the device while changing the current density. (a,b) Topological charge vs time during the injection process. (c,d) Topological charge vs time during the ejection process.

heating effects. A device geometry consisting of a two $10 \times 10\,\mu$m plates of thicknesses 300 nm and 500 nm was simulated with a triangular mesh generated by COMSOL. Ground and terminal surfaces were defined along the current direction with a heat exchange layer defined on the bottom at 250 K. The only other source of heat loss to the sample was defined as surface-to-ambient radiation with a surface emissivity of 0.8, where 1 is a perfect black-body. No significant differences were observed in the heating effects when reducing the surface emissivity. FeGe material parameters used to calculate current density distributions and Joule heating effects were defined as follows: electrical conductivity $\sigma = 4.55 \times 10^5\,\mathrm{S\,m^{-1}}$,[9] density, $\rho = 8220\,\mathrm{kg\,m^{-3}}$, heat capacity at constant pressure, $C_p = 400\,\mathrm{J\,kg^{-1}\,K}$,[10] and thermal conductivity, $\kappa = 80\,\mathrm{W\,m^{-1}\,K}$. The resulting simulations are summarised in Fig. 7.

From the simulated Joule heating, the maximum change in temperature across the sample is 4 mK (Fig. 7a) and does not depend on the current direction. A small heat gradient is

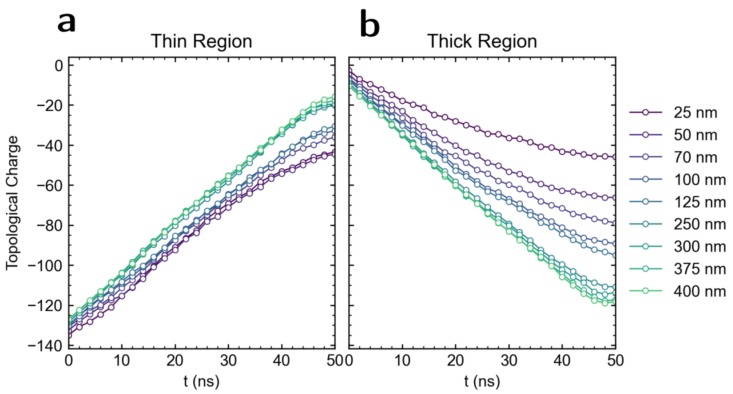

Figure 6: Z = 0 topological charge within each of section of the device while changing the thickness of the thinner section of the geometry. Injection is possible for all thicknesses however the number of skyrmions injected into the thicker region reduces with reducing thickness.

induced across the thickness of the sample due to the majority of the thermal conduction experienced from the bottom surface conduction. However, the 4 mK temperature rise and subsequent heat gradient is not sufficient to replicate the observed injection effects.

Injected Skyrmion Lifetimes

To gain some information on the metastable lifetimes of the injected skyrmions, we measured the intensity of the peaks every 5 seconds for 2500 seconds to determine if there was any significant reduction in the intensity over this period as an indicator of metastable decay. After roughly 20 minutes, we realigned the sample to account for thermal drift, subsequently measuring for another 20 minutes. The results at 255 K are indicated in Fig. 8. Fitting the data to an exponential decay as per

$$I(t) = I_0 \exp(-t/\tau), \tag{5}$$

where I_0 denotes the initial intensity and τ indicates the decay lifetime, we obtain a characteristic lifetime of $\tau = (670 \pm 4) \times 10^2$ s, approximately 18.5 hours.

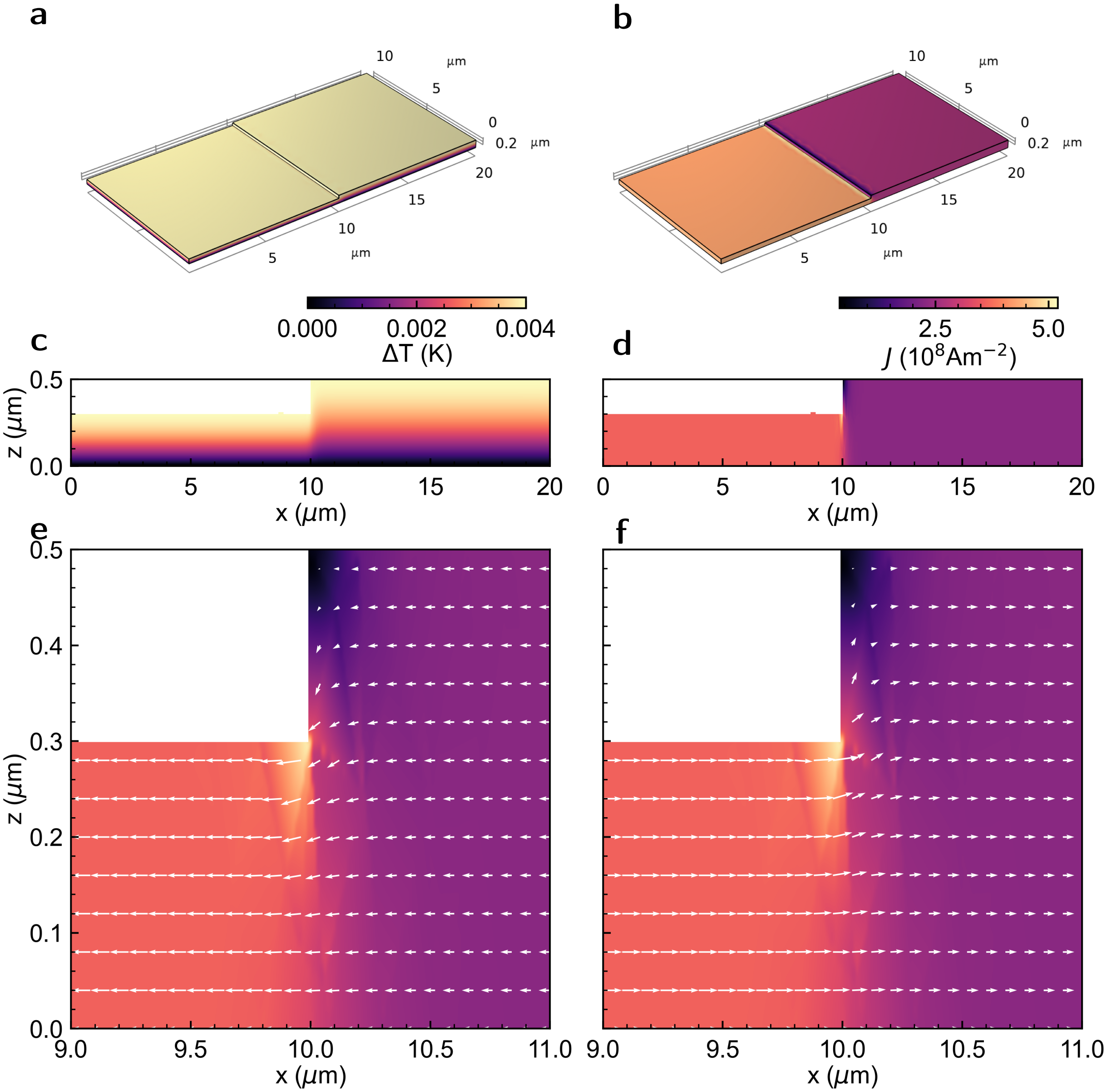

Figure 7: Heating and current density simulations: (a) Simulated Joule heating of the device structure. Note that these results are identical irrespective of the current direction. (b) Current density simulation indicated an amount of current bunching at the thickness barrier. y = 0 planes of both change in temperature and current density in (c) and (d) respectively. (e, f) y = 0 quiver map of the current density for both current polarities indicating equivalent current bunching.

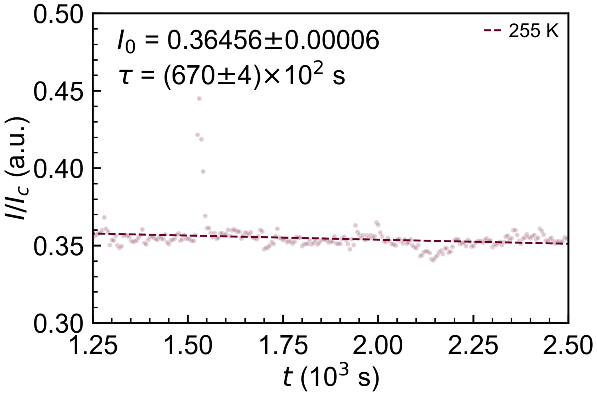

Figure 8: Plot of the intensity of the injected skyrmion scattering pattern as a function of time after waiting roughly 20 minutes to account for thermal drift.

Potential Device Applications

Here we discuss suggestions for potential applicability of this concept in device elements. Primarily, the thickness dependent properties of this concept allow the energetically expensive parameters (temperature and magnetic field) to remain constant during operation. This then allows an all electrical operation of the device structures.

Example applications include skyrmion reserviour computing and racetrack like schemes. In the former, the device can be maintained in the injection condition described in the main text where a reserviour of skyrmions exist within the thin region. During a writing operation involving an injection current pulse, a disordered array of skyrmions will be moved into the thicker region of the device in which any operations and readouts can be performed, after which an ejection pulse can be performed to reset the system without changing temperature or magnetic field. This provides numerous advantages within the neuromorphic computing regime where intrinsic disorder and damping within the reserviour are sought after properties to aid in the complexity required to perform specific operations without the need to train the network.

In the latter mentioned device, the injection element provides an important characteristic for the operability of a racetrack scheme. Under normal circumstances, if the entire racetrack

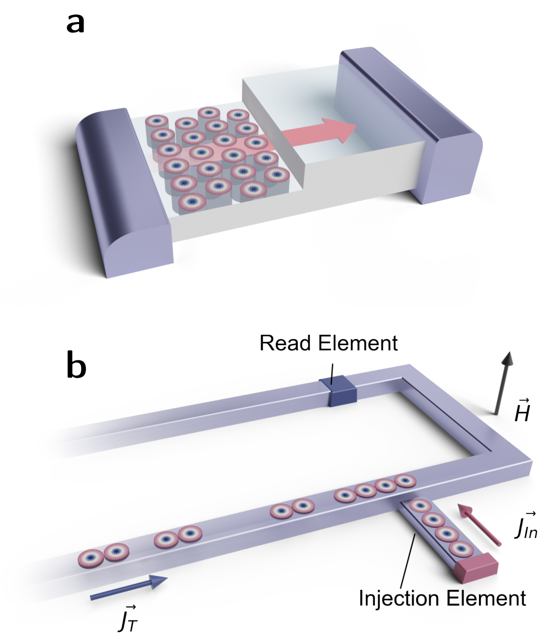

Figure 9: (a) Reserviour type devices utilising the injection mechanism. (b) Skyrmion racetrack scheme in which the injection mechanism provides the ability to write data in binary form.

were emersed in a global magnetic field, skyrmions would form in the entirety of the device, yielding no ability to detect the absence of a skyrmion, and therefore no ability to store binary data. With the injection mechanism, the entire device can still be maintained in a global magnetic field, and single skyrmions may be injected into the main track from a separate, thinner injector element. The racetrack can then be manipulated as required where the injector element provides the means to create the existence and absence of a skyrmion, thereby allowing binary data storage.

Supplementary Movies

- Movie S1: Animated GIF of simulated skyrmion injection process described in main text.
- Movie S2: Animated GIF of injection and ejection for a 300 nm/ 500 nm injector using

a current density of $3.32 \times 10^6\,\mathrm{A\,m^{-2}}$. In each of the following movies, the animation shows the integrated z-component of the magnetisation for both the thin and thick regions in the top panels and their corresponding SAXS signal in the bottom panels.

- Movie S3: Animated GIF of injection and ejection for a 300 nm/ 500 nm injector using a current density of $1.54 \times 10^7\,\mathrm{A\,m^{-2}}$
- Movie S4: Animated GIF of injection and ejection for a 300 nm/ 500 nm injector using a current density of $7.15 \times 10^7\,\mathrm{A\,m^{-2}}$
- Movie S5: Animated GIF of injection and ejection for a 300 nm/ 500 nm injector using a current density of $3.32 \times 10^8\,\mathrm{A\,m^{-2}}$
- Movie S6: Animated GIF of injection and ejection for a 300 nm/ 500 nm injector using a current density of $1.54 \times 10^9\,\mathrm{A\,m^{-2}}$
- Movie S7: Animated GIF of injection and ejection for a 300 nm/ 500 nm injector using a current density of $7.15 \times 10^9\,\mathrm{A\,m^{-2}}$
- Movie S8: Animated GIF of injection and ejection for a 300 nm/ 500 nm injector using a current density of $3.32 \times 10^{10}\,\mathrm{A\,m^{-2}}$
- Movie S9: Animated GIF of injection and ejection for a 300 nm/ 500 nm injector using a current density of $1.54 \times 10^{11}\,\mathrm{A\,m^{-2}}$
- Movie S10: Animated GIF of injection and ejection for a 300 nm/ 500 nm injector using a current density of $7.15 \times 10^{11}\,\mathrm{A\,m^{-2}}$
- Movie S11: Animated GIF of injection and ejection for a 300 nm/ 500 nm injector using a current density of $3.32 \times 10^{12}\,\mathrm{A\,m^{-2}}$
- Movie S12: Animated GIF of injection for thicknesses 25 nm/500 nm.
- Movie S13: Animated GIF of injection for thicknesses 50 nm/ 500 nm.

- Movie S14: Animated GIF of injection for thicknesses 70 nm/ 500 nm.
- Movie S15: Animated GIF of injection for thicknesses 100 nm/500 nm.
- Movie S16: Animated GIF of injection for thicknesses 125 nm/500 nm.
- Movie S17: Animated GIF of injection for thicknesses 250 nm/500 nm.
- Movie S18: Animated GIF of injection for thicknesses 300 nm/500 nm.
- Movie S19: Animated GIF of injection for thicknesses 375 nm/500 nm.
- Movie S20: Animated GIF of injection for thicknesses 400 nm/500 nm.

References

(1) Beg, M.; Pepper, R. A.; Fangohr, H. User interfaces for computational science: A domain specific language for OOMMF embedded in Python. *AIP Advances* **2017**, *7*, 056025.

(2) Beg, M.; Lang, M.; Fangohr, H. Ubermag: Towards more effective micromagnetic workflows. *IEEE Transactions on Magnetics* **2022**, *58*, 1–5.

(3) Donahue, M. OOMMF User's Guide, version 1.0. 1999.

(4) Bak, P.; Jensen, M. H. Theory of helical magnetic structures and phase transitions in MnSi and FeGe. *Journal of Physics C: Solid State Physics* **1980**, *13*, L881.

(5) Birch, M. T. et al. Real-space imaging of confined magnetic skyrmion tubes. *Nature Communications* **2020**, *11*, 1726.

(6) Zhang, S.; Li, Z. Roles of Nonequilibrium Conduction Electrons on the Magnetization Dynamics of Ferromagnets. *Physical Review Letters* **2004**, *93*, 127204.

(7) Turgut, E.; Stolt, M. J.; Jin, S. Topological spin dynamics in cubic FeGe near room temperature. *Journal of Applied Physics* **2017**, *122*, 183902.

(8) Iwasaki, J.; Mochizuki, M.; Nagaosa, N. Current-induced skyrmion dynamics in constricted geometries. *Nature Nanotechnology* **2013**, *8*, 742–747.

(9) Kanazawa, N.; Shibata, K.; Tokura, Y. Variation of spin–orbit coupling and related properties in skyrmionic system $Mn_{1-x}Fe_xGe$. *New Journal of Physics* **2016**, *18*, 045006.

(10) Wilhelm, H.; Leonov, A. O.; Rößler, U. K.; Burger, P.; Hardy, F.; Meingast, C.; Gruner, M. E.; Schnelle, W.; Schmidt, M.; Baenitz, M. Scaling study and thermodynamic properties of the cubic helimagnet FeGe. *Physical Review B* **2016**, *94*, 144424.
